# Identification of potential aryl hydrocarbon receptor ligands by virtual screening of industrial chemicals

**DOI:** 10.1007/s11356-017-0437-9

**Published:** 2017-11-10

**Authors:** Malin Larsson, Domenico Fraccalvieri, C. David Andersson, Laura Bonati, Anna Linusson, Patrik L. Andersson

**Affiliations:** 1 Department of Chemistry, Umeå University, SE-901 87 Umeå, Sweden; 2 Department of Earth and Environmental Sciences, University of Milano-Bicocca, Piazza della Scienza 1, 20126 Milan, Italy

**Keywords:** Virtual screening, Aryl hydrocarbon receptor, Industrial chemicals, Molecular descriptors, Structural similarity, Molecular docking

## Abstract

We have developed a virtual screening procedure to identify potential ligands to the aryl hydrocarbon receptor (AhR) among a set of industrial chemicals. AhR is a key target for dioxin-like compounds, which is related to these compounds’ potential to induce cancer and a wide range of endocrine and immune system-related effects. The virtual screening procedure included an initial filtration aiming at identifying chemicals with structural similarities to 66 known AhR binders, followed by 3 enrichment methods run in parallel. These include two ligand-based methods (structural fingerprints and nearest neighbor analysis) and one structure-based method using an AhR homology model. A set of 6445 commonly used industrial chemicals was processed, and each step identified unique potential ligands. Seven compounds were identified by all three enrichment methods, and these compounds included known activators and suppressors of AhR. Only approximately 0.7% (41 compounds) of the studied industrial compounds was identified as potential AhR ligands and among these, 28 compounds have to our knowledge not been tested for AhR-mediated effects or have been screened with low purity. We suggest assessment of AhR-related activities of these compounds and in particular 2-chlorotrityl chloride, 3-*p*-hydroxyanilino-carbazole, and 3-(2-chloro-4-nitrophenyl)-5-(1,1-dimethylethyl)-1,3,4-oxadiazol-2(3H)-one.

**Electronic supplementary material:**

The online version of this article (10.1007/s11356-017-0437-9) contains supplementary material, which is available to authorized users.

## Introduction

2,3,7,8-Tetrachlorodibenzo-*p*-dioxin (2378-TCDD) is a persistent organic pollutant that is known to induce several toxicological outcomes ranging from acute syndromes such as chloracne in humans to severe long-term adverse health effects, including immune response-related effects, cancer, and reproductive disorders (Geyer et al. 2002; Safe 1990; Sorg et al. 2009; Van den Berg et al. 1998; Van den Berg et al. 2006). AhR plays a central role in the toxicological outcomes of 2378-TCDD, which is the most potent known ligand of AhR (Denison and Nagy 2003). In addition to dioxins and dioxin-like chemicals, AhR can bind to and be activated by a number of diverse natural, endogenous, and synthetic compounds (Denison and Nagy 2003). The AhR signaling pathway is also known to cross talk with the estrogen receptor pathway, which implies that AhR-activating compounds can result in endocrine disruption (Klinge et al. 1999; Ohtake et al. 2011; Swedenborg and Pongratz 2010). Furthermore, activation of AhR has also been linked to many immune response processes that are associated with numerous diseases (Bessede et al. 2014; Boule et al. 2014; Frawley et al. 2014; Heilmann et al. 2010; Hochstenbach et al. 2012; Stølevik et al. 2013; Winans et al. 2011). Thus the identification and regulation of chemicals that induce AhR-related pathways is a critical human health and environmental issue. Non-animal testing methods, including in vitro and in silico methods, are promoted in the European chemical legislation REACH as ways to identify and prioritize chemicals for further testing (REACH 2007). In the USA, the Tox21 and the ToxCast programs were initiated to identify hazardous chemicals by using high-throughput screening approaches, including large batteries of in vitro assays (Berg et al. 2015; Dix et al. 2007; Filer et al. 2014; Judson et al. 2010; Oki and Edwards 2016; Richard et al. 2016; Rotroff et al. 2010; Rotroff et al. 2013; Tox21; ToxCast). In the Tox21 program, 10,486 chemicals were screened for AhR mediated activity, of which 1063 chemicals were identified as AhR agonists in the human cell-based HepG2-AhR-luciferase reporter gene assay (NCBI).

Virtual screening is frequently used in medicinal chemistry where in silico methodologies are used to evaluate large compound libraries for potential associations with a well-defined target, usually a protein (Gohlke and Klebe 2002). To evaluate the virtual screening output, the hits are often verified by competitive binding assays and/or other in vitro assays (Kouskoumvekaki et al. 2013). Virtual screening is based on the structural data of known ligands of the studied target (ligand-based screening) and/or characteristics of the target protein (structure-based screening) (Ai et al. 2015; AlQudah et al. 2016; Bisson et al. 2009; Cross et al. 2012; Kitchen et al. 2004; Spyrakis and Cavasotto 2015; Swann et al. 2011; Svensson et al. 2011; Xie et al. 2014). Ligand-based information includes structural fingerprints and specific chemical properties, while structure-based virtual screening is based on interactions between candidate ligands and the receptor as evaluated by molecular docking methods (Kitchen et al. 2004; Spyrakis and Cavasotto 2015). Currently, no X-ray crystal structure of the AhR ligand binding domain (LBD) is available, and thus homology models have been developed to enable detailed studies of ligand interactions (Bisson et al. 2009; Lo Piparo et al. 2006; Motto et al. 2011; Pandini et al. 2009). The binding free energies of docking poses obtained from a homology model of the AhR LBD have been shown to correlate well with the experimentally derived competitive binding affinities of 14 polychlorinated dibenzo-*p*-dioxins (PCDDs), including 2378-TCDD, indicating that this homology model can be used for virtual screening purposes (Motto et al. 2011).

The aim of this study was to use virtual screening to identify new potential AhR ligands among a set of commonly used industrial chemicals. A virtual screening protocol was developed based on structural information from AhR binders that have been shown to induce AhR-related responses and on information from a rat AhR homology model (Motto et al. 2011). Ligand similarities were determined by structural fingerprints and by nearest neighbor analysis based on 2D-descriptors. Fingerprint-based approaches identify similar substances based on their molecular sub-structures, while nearest neighbor analysis identifies similarities in chemical and structural properties. Protein-ligand interactions were evaluated using the binding free energies of the docking poses. When creating the protocol, we used the three screening steps in parallel because multiple scoring and data fusion have proven to be more robust than, and often outperform, a single virtual screening method (Baber et al. 2006; Swann et al. 2011; Svensson et al. 2011; Willett et al. 1998). To analyze the developed virtual screening protocol, we compared the results from each parallel method with data on AhR activation from the Tox21 database (NCBI). Chemicals top-ranked by at least two of the three parallel methods were identified as potential AhR ligands. Data on AhR-mediated effects of these chemicals were searched for in the open scientific literature and the Tox21 database (NCBI).

## Materials and methods

### Datasets

The literature search focused on finding compounds that activate AhR in rat or mouse cell assays, i.e., ethoxyresorufin-*O*-deethylase (EROD) activity or dioxin-responsive chemically activated luciferase expression (DR-CALUX) assays. These compounds are hereafter called “AhR modulators.” The search was performed in SciFinder (2014-09-25) using the following delimiters: “CALUX dioxin”; “EROD” with refinement “relative potency, not soil, not sediment, not contamination, not diet”; and “EROD” with refinement (a) “in vitro”, (b) “luciferase dioxin AhR”, and (c) “luciferase AhR.” Reported data from the EROD and CALUX assays showed high correlation, and we thus decided to merge these data (Supporting Information).

A second database was derived with compounds that bind to AhR, here called the “AhR binders,” that were selected based on a reported half-maximum inhibition concentration (IC_50_), inhibition constant (*K*_i_), or dissociation constant (*K*_d_) at or below 10 μM as measured in competitive binding assays using labeled 2378-TCDD in rat or mouse cell systems (Dataset S2). The binding data were obtained from SciFinder (2014-09-25) using the delimiter “Ah receptor” with refinement “agonist affinity” and “Ah receptor competitive.” We also used the available ligand binding data from the studies obtained in the literature search for AhR modulators. In addition, compounds were defined as binders if 50% of the labeled 2378-TCDD was displaced at compound concentrations at or below 10 μM (Hu et al. 2007). The threshold of 10 μM was adopted to avoid false positives as in vitro data above that limit might be erroneous. Several classical AhR ligands are very hydrophobic and have very limited water solubility which increases the experimental uncertainty in that concentration range. Applying this threshold also means that we use a safety factor of approximately 1000 in relation to levels reported in humans of AhR ligands. For example, very high levels of PCBs in human blood (up to 15 nM) have been reported for populations in Eastern Slovakia (Petrik et al. 2006).

**Fig. 1:**
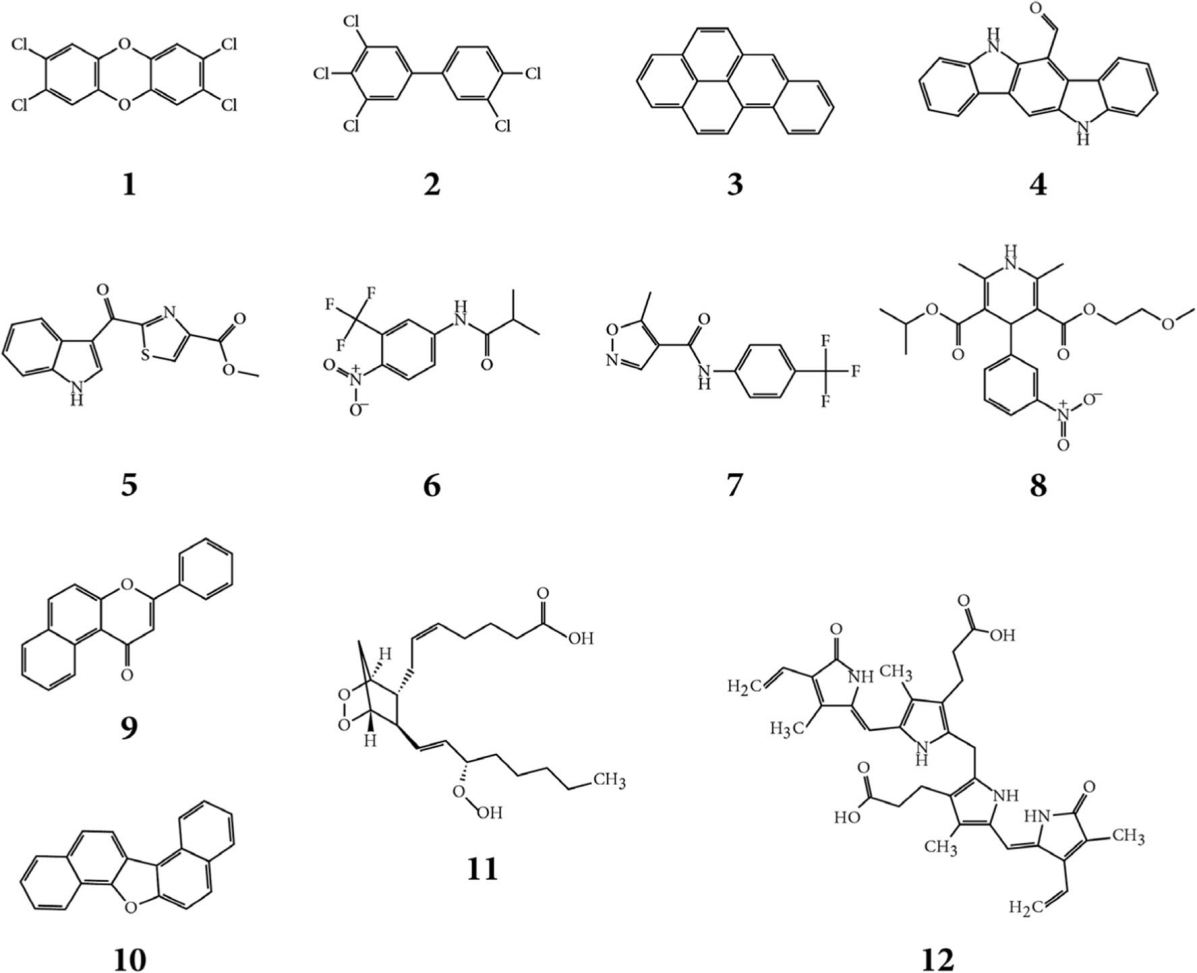
Molecular structures of selected compounds that have shown AhR-mediated effects in vitro (AhR modulators, **1**–**12**) and for which some have been shown to competitively bind to AhR (AhR binders, **1**–**9**). Numbers **1**, **2**, **3**, and **9** are examples of typical AhR ligands, i.e., small, rigid, aromatic compounds. Numbers **4**, **5**, **6**, **7**, and **8** are atypical AhR ligands, i.e., they are rather flexible aromatic compounds containing additional functional groups and atom types compared to the typical AhR ligands. **1**) 2,3,7,8-tetrachloro-dibenzo-*p*-dioxin (2378-TCDD), **2**) 3,3′,4,4′,5-pentachloro-biphenyl (PCB 126), **3**) benzo-a-pyrene (BaP), **4**) 6-formylindolo[3,2-b]carbazole (FICZ), **5**) 2-(1’H-indole-3′-carbonyl)-thiazole-4-carboxylic acid ester (ITE), **6**) flutamide, **7**) leflunomide, **8**) nimodipine, **9**) beta-naphthoflavone (BNF), **10**) dinaphtho[1,2-b;1′2’-d]furan (DNF), **11**) prostaglandin G2, **12**) bilirubin

The third analyzed data set covered an inventory of high and low production volume chemicals (H/LPVCs) (Rannar and Andersson 2010), i.e,. the “industrial chemicals.” Compounds including atoms Al, As, Ba, Bi, Cd, Co, Cr, Mn, Ni, Pb, Sb, Sn, Sr, Ti, or Zr were removed due to the lack of MMFF94x force field parameters (Halgren 1999) and the final H/LPVC database consisted of 6445 industrial chemicals. Chemical Abstracts Service (CAS) numbers were used to identify each chemical. The molecular structures of all compounds discussed are given in Figs. 1, 6, and S7 and in Tables S3–S5.

### Chemical structures and molecular descriptors

The substances in the AhR modulators set were characterized using 68 calculated 2D (Rannar and Andersson 2010) and 18 3D descriptors (Table S1). These 86 descriptors were used for creating an overview of the structural and chemical variation of the AhR modulators. For the 3D descriptors, the chemical structures were energy minimized using the MMFF94x force field and Austin Model 1 (AM1) (Dewar et al. 1985; Halgren 1999; Molecular Operating Environment 2012.10) prior to calculations of force field-based and quantum chemistry-based descriptors, respectively. The descriptors were obtained using MOE version 2012.10 (Molecular Operating Environment 2012.10), except for one shape descriptor, DistMax (the longest distance in Angstrom (Å) between two atoms in the molecule), which was calculated based on the AM1 3D-coordinates using MATLAB version R2012a (MATLAB R2012a). Only 2D descriptors were used for the virtual screening, and therefore the structures of the H/LPVC database were solely characterized by the 68 2D descriptors (cf. the 2D descriptors calculated for the AhR modulators). The structures were pre-treated, i.e., strong acids were deprotonated, strong bases were protonated, and counter ions and duplicates were removed, and structures were energy minimized prior to calculations of the molecular descriptors using MOE (Molecular Operating Environment 2012.10). The descriptors covered features to describe hydrophobicity, polarizability, reactivity, aromaticity, flexibility, size, and shape. For example, the hydrophobicity of the compounds was reflected by the octanol-water partition coefficient (logP) and the reactivity of the compounds by molecular orbital energies such as the highest occupied molecular orbital (HOMO) and the lowest unoccupied molecular orbital (LUMO) energies and the difference between these energies (GAP). Polarity was provided by the implementation of the Gasteiger and Marsili partial equalization of orbital electronegativities (PEOE) method, which describes the polarity of the molecular surface (the van der Waals surface) based on atomic partial charges (PEOE_VSA) (Molecular Operating Environment 2012.10). The shape of the molecules was estimated by connectivity indices, such as the Balaban distance connectivity index J (balabanJ) and kappa shape indices (Kier1, Kier2, and Kier3) (Balaban 1979; Hall and Kier 1991) as well as by the ratios of the 3D descriptors pmi1, pmi2, and pmi3, which reflect relations in the width, height, and length of the molecule (Sauer and Schwarz 2003). Many known AhR ligands are aromatic, and thus descriptors related to this property were included, such as the numbers of aromatic bonds and rings.

### Techniques used for multivariate analysis and virtual screening

#### Principal component analysis

Principal component analysis (PCA) is a projection method where the largest variation in the data is extracted by creating new latent orthogonal variables. Here, each object is a molecule and the variables are the molecular descriptors. The first principal component (PC) shows the largest variation in the data, i.e., the largest variation between the molecules based on their chemical and structural characteristics. The second PC is orthogonal to the first one and reflects the second largest variation and so on. Each PC is defined by score values representing the locations of the molecules in the multivariate space (using projections to the latent variable) and by loading values that indicate which descriptors are responsible for the distribution seen in the score values. PCA was used for the analysis of the AhR modulators and was used in the initial filtration step and in the nearest neighbor analysis of the virtual screening procedure (“Euclidean distances in the chemical property space” section). To evaluate the PCA model, the following statistical measures were used: Distance to the Model (DModX), eigenvalues, the explained variation in each PC (R^2^X), and Hotelling statistics (Hotelling’s T^2^ range at the 95% confidence level). DModX and Hotelling’s T^2^ were used to assess the applicability domain of the studied compounds and to detect outliers among the compounds (SIMCA 13.0). A PC was considered significant if it had an eigenvalue equal or above 2. The PCA was performed with SIMCA version 13.0 (SIMCA 13.0).

#### Ligand-based similarity techniques

##### Tanimoto coefficients

Molecular ACCess System (MACCS) fingerprints were used to define the fragments for the fingerprinting (MACCS Keys). MACCS contains a total of 166 predefined structural keys that correspond to various fragments that form binary fingerprints of the molecules. The fingerprints between pairs of molecules were compared using the Tanimoto coefficient (TC) that describes the similarities between the binary strings according to

1 $$ TC=\frac{n_{SAME}}{n_A+{n}_B-{n}_{SAME}}\kern0.5em $$

where n_SAME_ is the number of identical fragments in molecule A and molecule B and n_A_ and n_B_ are the numbers of fragments in molecules A and B, respectively (Willett et al. 1998). A TC of 0.60 was set as the cut-off for calling molecule A and B similar in this study (Supporting Information). The TCs were calculated in MOE version 2012.10 (Molecular Operating Environment 2012.10).

##### Euclidean distances in the chemical property space

The distance between molecules in a defined chemical property space (here defined by latent variables from the PCA) can be used as a measure of molecular similarity. Here, the Euclidean distance (ED) between two molecules based on the Cartesian coordinates of the latent variables from PCA was calculated according to:

2 $$ {ED}_{pq}=\sqrt{{\left({q}_1-{p}_1\right)}^2+{\left({q}_2-{p}_2\right)}^2+\dots +{\left({q}_n-{p}_n\right)}^2} $$

where *p* and *q* are two molecules, *p*_1_, *p*_2_, …, *p*_*n*_ are the Cartesian coordinates given by the score values of the 1 to *n* PCs for molecule *p*, and *q*_1_, *q*_2_, …, *q*_*n*_ are the Cartesian coordinates given by the score values of the 1 to *n* PCs for molecule *q* (Willett et al. 1998). EDs were used to locate closest neighbors to each of the AhR binders, and the cut-offs for the EDs differed according to the scaling of the data for the PCAs used in the screening steps. The ED cut-offs were set based on the point at which the structures no longer shared the same number of rings and/or similar functional groups in the same positions as in the AhR binders. An ED of 1.5 was used in the initial filtration step to provide structurally similar compounds to a few structurally diverse AhR binders. For the nearest neighbor analysis in the parallel virtual screening step, the ED was set to 5.0 and a maximum of ten neighbors was kept for each AhR binder. The rationale for the much smaller ED cut-off in the initial filtration step was that the descriptors (except those already log-transformed) were log-transformed prior to analysis to normalize their distribution and to minimize the influence of extreme values (Rannar and Andersson 2010). More information on the cut-off procedure is given in the Supporting Information.

#### Docking protocol and evaluation

A previously generated homology model of the LBD of the rat AhR (Motto et al. 2011), which was derived from the template structures of three HIF-2α PAS-B domains in complexes with artificial ligands (Key et al. 2009; Scheuermann et al. 2009), was used to study the molecular interactions between the potential ligands and the LBD. The docking procedure was based on a previously developed protocol for docking to homology models (Motto et al. 2011) and included refinement of the model containing a template ligand (THS-017 (Key et al. 2009)) by energy minimization with the MacroModel program included in Maestro (Schrödinger Release 2014b–3: MacroModel), docking using the Glide 6.2 SP program (Friesner et al. 2004; Schrödinger Release 2014a–3: Glide), and refinement and rescoring of the docking poses with the generalized Born/surface area (MM-GBSA) molecular mechanics method as implemented in the Prime software (Schrödinger Release 2014c–3: Prime). Compared to the previously adopted ensemble-docking protocol (Motto et al. 2011), only one receptor conformation was selected for docking in this work so as to reduce the computational costs. The receptor grid for docking was centered on the THS-017 ligand, and docking was performed within a 12 Å distance from the ligand position (Key et al. 2009; Motto et al. 2011). Tautomerisation and protonation at pH 7.4, as well as stereoisomerism, were generated and used for the studied ligands using the program LigPrep in Maestro. The ten highest-ranked docking poses of each ligand stereoisomer, according to the GlideScore SP scoring function, were rescored with the Prime MM-GBSA method that allows for estimation of the binding free energy (ΔG_bind_) between the compounds and AhR, which accounts for the interaction energies and desolvation effects that occur upon complex formation. This method yielded ΔG_bind_ values for the docking poses of PCDD/Fs and PAHs (Motto et al. 2011; Piskorskapliszczynska et al. 1986; Safe 1990) that correlate well with experimental IC_50_ values. In the rescoring procedure, the ligands and protein residues within 8.0 Å from the ligand were energy minimized while the remaining residues were kept fixed. The AhR–ligand complexes with the lowest ΔG_bind_, including one pose of a specific stereoisomer of each ligand, were analyzed further. We estimated a cutoff based on the ΔG_bind_ values of the 65 known AhR binders (one failed in the docking procedure) which had an average ΔG_bind_ of −112.5 kcal/mol. The industrial chemicals that had a ΔG_bind_ value within one standard deviation of the average ΔG_bind_ (−99.3 kcal/mol) of the 65 known binders were considered more likely to be potential AhR binders than those with higher ΔG_bind_ values. This selection became the final enrichment set from the molecular docking. More information on the cutoff procedure is given in the Supporting Information. Docked and rescored ligands were classified based on MACCS fingerprint descriptors using a hierarchical clustering model based on Tanimoto coefficients (Eq. 1) (Canvas 2.5) and a cluster linkage method based on the weighted average intra-cluster and inter-cluster distances (Lance and Williams 1967) with a beta value of 0.25. Hydrogen bonds, halogen bonds, and aromatic π–π and hydrogen–π bonds between AhR and ligands were mapped using MOE (Molecular Operating Environment 2012.10) with a distance cut-off of 4.5 Å and a maximum interaction energy of −0.5 kcal/mol, taking atom pair distance and directionality into account.

### Evaluation study of the virtual screening protocol

To analyze the results from the parallel screening methods, we searched the Tox21 database for data on AhR-mediated effects. The 429 compounds, resulting from the initial filtration step, were searched for in the Tox21 Concentration Browser (https://ntp.niehs.nih.gov/sandbox/tox21-curve-visualization/) with CAS numbers as compound identifiers. To minimize influence of impurities, we only included chemicals that had the highest purity rate (> 90%, i.e., classified as A and Ac) (Dataset S8). Moreover, chemicals that were stated to be both active and inactive (multiple experiments) were excluded. The performance of our screening approach was evaluated by calculating the accuracy, sensitivity, and specificity (Table S9) (Mannhold et al. 2009) using the classification (active agonist/inactive) from the Tox21 data (NCBI).

We performed a literature search for AhR-mediated effects for the 41 compounds that were jointly identified as potential AhR ligands by at least 2 parallel methods in the virtual screening procedure (Dataset S7, Table S5). The search was performed in SciFinder (2016-03-16) using these compounds’ CAS numbers and limiting the search to the options “Adverse effect, including toxicity” and also retrieving “Additional related references, e.g., activity studies, disease studies.” This resulted in an individual list of references for each compound, on which the following SciFinder filters were applied: (a) “CYP1A1,” (b) “CYP1A,” (c) “CYP1,” and (d) “AhR.”

## Results and discussion

### Chemical characterization of AhR modulators

The AhR modulator database includes a range of small and halogenated aromatic compounds and polycyclic aromatic compounds that are known to induce AhR-mediated effects (Dataset S1). Examples of typical AhR modulators, besides 2378-TCDD, are 3,3′,4,4′,5-pentachlorobiphenyl (PCB 126), benzo-a-pyrene (BaP), and beta-naphthoflavone (BNF) (Fig. 1). The database also included endogenous rigid aromatics such as 6-formylindolo[3,2-b]carbazole (FICZ) and dinaphtho[1,2-b;1′2’-d]furan (DNF), flexible aromatic compounds such as 2-(1’H-indole-3′-carbonyl)-thiazole-4-carboxylic acid ester (ITE), and large, complex, and flexible compounds like prostaglandin G2 and bilirubin.

**Fig. 2:**
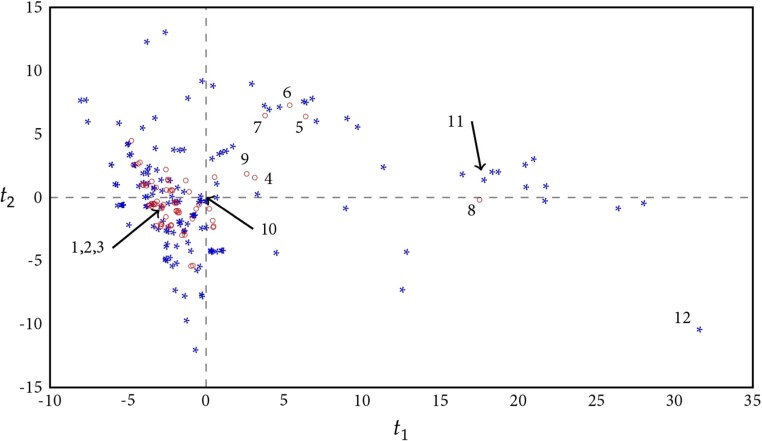
The score plot of the first and second principal components of the 214 AhR modulators identified in the literature based on 68 2D-descriptors and 18 3D-descriptors. The 66 known AhR binders are shown as red circles, and the remaining AhR modulators are shown as blue stars. The numbers refer to the molecular structures given in Fig. 1

PCA analysis of the structural variation of the 214 AhR modulators resulted in 5 significant PCs explaining 48, 17, 13, 5, and 4% of the variation in the data, respectively. By studying the first three components, a clear separation into four clusters emerged, including (1) halogenated aromatics, (2) polycyclic aromatic hydrocarbons (PAHs), (3) natural products, and (4) endogenous substances (Fig. S3). The largest cluster in the score plot included the majority of the halogenated aromatic compounds, and this showed that most of the AhR modulators are structurally similar (Fig. 2). The first PC described the difference between compounds with regards to size and surface characteristics, the second PC described the difference between compounds with regards to hydrophobicity, and the third PC described the difference between compounds with regards to density; the numbers of rings, aromatic bonds, and halogens; GAP; and the shape/branching index balabanJ. The fourth PC separated compounds based primarily on their LUMO energy, the number of rotatable bonds, and the number of rings in relation to the number of atoms. The fifth PC was related to variations in molecular shape described by the ratios of shape descriptors, including the length, width, and height of the molecules (pmi2/pmi1, pmi3/pmi1, npr2 = pmi2/pmi3). The set of AhR modulators also included roughly 40 structurally diverse compounds of which many were more flexible and less hydrophobic compared to most AhR modulators. As seen in the PCA (Fig. 2), the AhR modulators cover a large variation in structural characteristics, and it might be questioned if all of these compounds actually bind to AhR (Denison and Nagy 2003; Denison et al. 2011). The development of the virtual screening protocol was therefore based on known AhR binders, and we identified 66 compounds in the literature that have AhR binding data (Dataset S2, Fig. 2).

### Virtual screening of industrial chemicals

**Fig. 3:**
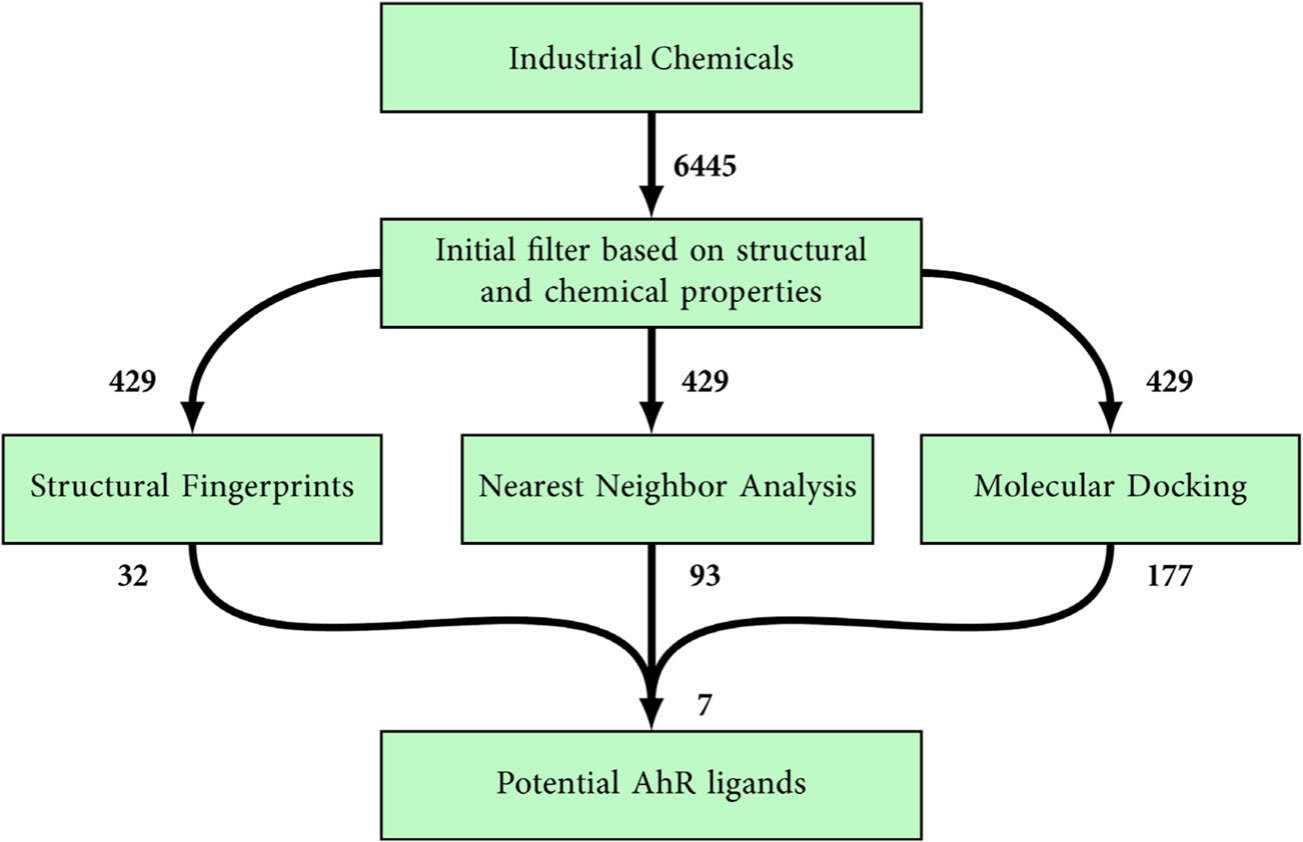
The flowchart of the virtual screening protocol for identifying AhR ligands among a set of 6445 industrial chemicals. The numbers next to the boxes correspond to the total number of compounds remaining at each step. The steps included an initial filter using principal component analysis followed by three parallel steps consisting of similarity measurements based on the 66 known AhR binders (nearest neighbor analysis and structural fingerprints) and molecular docking with an AhR homology model

The virtual screening procedure consisted of an initial filtration step followed by three parallel enrichment steps, including two ligand-based methods (nearest neighbor analysis and structural fingerprints) and one structure-based method (molecular docking) (Fig. 3).

#### Initial filtration based on structural and chemical properties

In order to identify potential AhR binders among the compounds in the H/LPVC database, we used an initial filtration step based on PCA. A total of 330 H/LPVCs were identified by PCA within the applicability domain of the 66 AhR binders. FICZ, ITE, flutamide, leflunomide, and nimodipine were identified as being outside the domain of the PCA model, and a parallel filtration step was introduced to cover substances with similar structural characteristics as these five compounds. This procedure was based on a PCA including mentioned 5 compounds and the 6445 compounds in the H/LPVC database, and it yielded an additional set of 99 industrial chemicals that are referred to here as atypical potential AhR binders. These compounds include, in general, those with a larger structural variation compared to the remaining 330 compounds, which are referred to as typical AhR binders (Fig. 1; 1–3 and 9). In summary, the filtration step resulted in 429 industrial chemicals that were further processed in the 3 parallel virtual screening procedures (Fig. S6).

#### Ligand-based methods

The enrichment based on the nearest neighbor analysis and the structural fingerprints of the 429 industrial chemicals (“Initial filtration based on structural and chemical properties” section) yielded 93 and 32 compounds, respectively (Fig. 3). The 93 compounds of the nearest neighbor analysis (Dataset S5) were identified close to known AhR binders in the chemical property space. These compounds typically included two or three aromatic rings, and 29 of the 93 compounds were halogenated and 8 of them had chlorine atoms in lateral positions on both outer rings, thus resembling the most potent AhR agonist, 2378-TCDD. These eight compounds typically included an oxygen, nitrogen, or sulfur connecting the halogenated aromatic rings, for example 5-chloro-2-(2,4-dichlorophenoxy)aniline (56966-52-0), tetradifon (116-29-0), and 2-(4-chlorophenoxy)-5-(trifluoromethyl)aniline (349-20-2). Similar structures, such as polychlorinated diphenyl sulfides, have been shown to activate mouse AhR in the H4IIE-luc activation assay (Zhang et al. 2016). In addition, 14 of the compounds were polycyclic and as such resembled PAHs, i.e., they had 3–5 phenyl rings and typically had no substituents on the rings. Eight of these 14 compounds had fused rings similar to the known potent PAHs with nitrogen or a carbonylic carbon in the phenyl rings, for example 12H-phthaloperin-12-one (6925-69-5). The aromatic rings were halogenated in two cases—3-bromobenz[de]anthracen-7-one (81-96-9) and 3,9-dibromo-7H-benz[de]anthracen-7-one (81-98-1). Similar molecules to the last two compounds have been shown to activate AhR in yeast cells (Ohura et al. 2007). Unlike the other polycyclic compounds, six compounds had non-fused rings and typically had three rings connected by a central phosphorous atom, for example triphenylphosphine (603-35-0). There were 46 neighbors for the AhR binder BNF, which shared hits with PAHs but also had unique neighbors, including naphthalene-like compounds with two fused rings and carbonylic functionalities, for example 2-(2-quinolyl)-1H-indene-1,3(2H)-dione (83-08-9). The remaining 50 compounds (of the 93) were similar to the atypical AhR binders (Fig. 1; 4–8). The atypical AhR binder FICZ, which is structurally quite rigid similar to the more typical binders, was identified close to 2,2′-thiophene-2,5-diylbis(benzoxazole) (2866-43-5) and 5,12-dihydroquino[2,3-b]acridine-7,14-dione (1047-16-1). ITE and leflunomide were found close to over 70 compounds. The ten closest neighbors of ITE all had hydrogen acceptor atoms at similar positions. For instance, 1,8-dihydroxy-4,5-bis(methylamino)anthraquinone (56524-76-6) has hydrogen acceptor atoms situated pair-wise in the middle and at both ends of the structure, which is similar to ITE (Table S3).

The 32 compounds identified using structural fingerprints (Dataset S4) had a similar share of halogenated compounds (approximately one third) as the 93 compounds identified by the nearest neighbor analysis. However, only 4 of these 32 compounds had chlorine atoms in lateral positions on both outer rings, i.e., resembling 2378-TCDD. The compounds identified by structural fingerprints included 12 halogenated aromatic compounds, and of the 32 compounds 15 were similar to typical AhR binders (halogenated aromatics, PAHs, and beta-naphthoflavone) and 17 were similar to atypical AhR binders. The three industrial chemicals nisoldipine (63675-72-9), bis(isopropyl)naphthalene (38640-62-9), and 4-bromo-2-fluoro-1,1′-biphenyl (41604-19-7) were very similar to known AhR binders (TC above 0.8). The highest similarity (TC = 0.86) was obtained for nisoldipine, which has the same core structure as nimodipine but different substituents. This compound was identified as an agonist in a human cell-based HepG2-AhR-luciferase reporter gene assay (NCBI). None of the identified 32 compounds (i.e., having TC ≥ 0.60) were similar to ITE or any of the PCDFs, unlike what was seen in the nearest neighbor analysis. However, the structural fingerprint-based method identified isoxazoles, one acidic isoflavone, and diphenyl-azo compounds with ester functionalities that were not identified in the nearest neighbor analysis. Three isoxazole acyl chlorides were found, which are used as precursors in the synthesis of antibiotics, e.g., cloxacillin (Gujral 2014; Li et al. 2011). Isoflavones with certain substitution patterns on their rings have been shown to induce luciferase activity in mouse H1L6.1c2 cells (Wall et al. 2012), and the isoflavone identified by the structural fingerprint approach, 3-methylflavone-8-carboxylic acid (3468-01-7), is a metabolite of the urinary incontinence drug flavoxate (Zaazaa et al. 2015). The azo-compounds, such as 2-[[4-[(2-cyano-3-nitrophenyl)azo]-m-tolyl](2-acetoxyethyl)amino] ethyl acetate (66882-16-4) (Arnold et al. 2012), are used as textile dyes.

#### Structure-based method

**Fig. 4:**
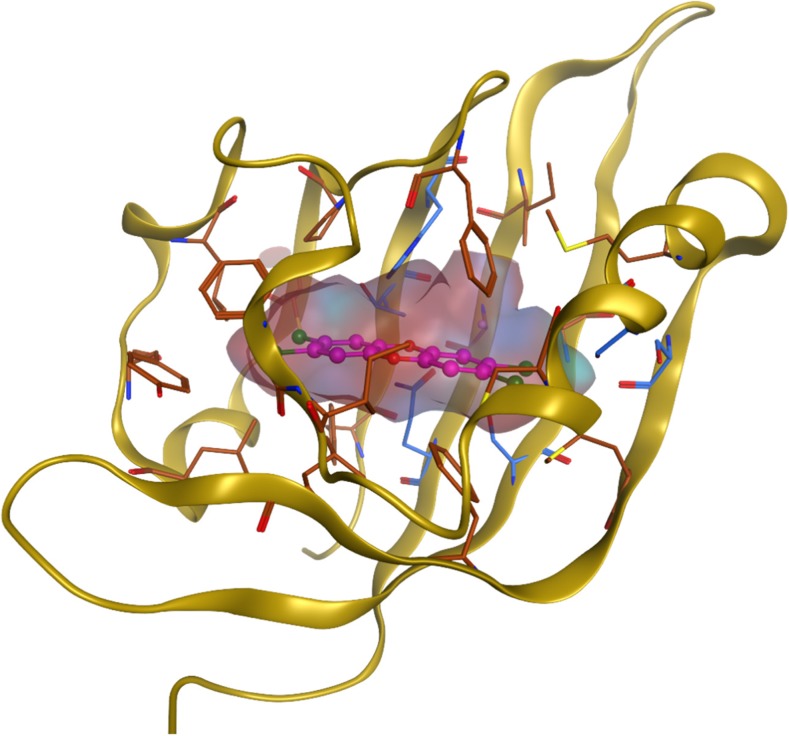
Cartoon representation of the AhR homology model showing the rather small and buried cavity occupied by the docked 2,3,7,8-tetrachlorodibenzo-*p*-dioxin. The cavity is mostly hydrophobic (depicted as brown sticks) but has a few polar amino-acid residues (depicted as blue sticks)

The 66 previously established AhR binders from the literature and the 429 industrial chemicals remaining after the initial filtering step were docked to a homology model of the AhR LBD (Motto et al. 2011). The AhR homology model used for the molecular docking shows a tunnel-shaped and buried ligand-binding cavity (Fig. 4). Out of the 23 residues flanking the binding site, 10 residues with hydrophobic side chains (Ala, Leu, Ile, Pro, Phe, Tyr) form 3 patches of hydrophobic surface covering a large proportion of the binding site surface. Eight amino acids line the site with O, N, or S atoms that can participate in hydrogen or halogen bonds. Analysis of the docking of the established AhR binders revealed that few molecules bonded to AhR with classical hydrogen bonds (O–H or N–H to O or N) even though a majority of the ligands contained a heteroatom with hydrogen bonding capability. Petkov et al. (2010) suggested that the heteroatom(s) present at the center of the PCDD/F structures constitutes a nucleophilic site of importance for binding to AhR. Another important feature stressed by these authors was the significance of having electrophiles on both sides of the PCDD/F molecule, i.e., lateral halogens. In our study, chlorine- or bromine-containing ligands frequently halogen bonded to oxygen (Thr287 (OH), Ala332, His330, Ser334 (OH)) or to sulfur (Cys298, Cys331, Met346, Met338). A few compounds interacted with the aromatic residue His289 through aromatic stacking (23479-PeCDF and 124678-HxCDF) or hydrogen–arene interactions (PCB77, PCB157, and 3-methylcholanthrene). This agrees well with the SARs presented by Petkov et al. (2010) where aromatic stacking was found to be of greater importance for PCBs and PAHs compared to PCDD/Fs. However, in our study, only 35% of the known binders participated in these specific interactions with AhR, and thus other factors, such as shape complementarity, van der Waals interactions, and desolvation effects were found to be important for most binders. From the cutoff based on ΔG_bind_ of the top-ranked poses, 177 of the 429 potential binders identified in the initial filtration step were singled out as more probable AhR binders than the remaining 252 chemicals. The 177 industrial chemicals (Dataset S6) had a relatively higher proportion of specific interactions with AhR compared to the docked and rescored known AhR binders, and 69% had at least one hydrogen bond, 11% had halogen bonds, and 39% had aromatic interactions. One third of the 177 industrial chemicals were halogenated, and most of these contained one or two halogen atoms.

The potential binders were assessed for their structural similarity to the known binders using a hierarchical clustering based on fingerprint descriptors, and the resulting clusters are presented in Fig. S7. Clusters 1, 2, 4, and 5 only contained known typical binders, and five clusters (3, 14, 15, 16, and 35) contained at least one known binder. A total of 153 compounds were identified in the remaining 26 clusters, and some clusters included small aromatic and often halogenated compounds (e.g., 20, 21, and 31), but the majority contained flexible and hydrophilic compounds with fused or nonfused aliphatic and aromatic rings (e.g. 7, 8, 9, and 25). The five clusters with at least one binder consisted of small aromatic, often halogenated, compounds, for example 2-chlorotritylchloride (42074–68-0) (Cluster 3). A unique feature captured by the molecular docking was the steroid ring structures. Examples of such chemicals were cholic acid (81-25-4) and ethisterone (434-03-7) (Cluster 7). Cholic acid is a bile acid with low water solubility (Moroi et al. 1992) indicating that a hydrophobic environment would suit it well, and it has similarities with the endogenous compound lipoxin 4A. Both compounds are acidic, flexible, and contain three hydroxyl groups, and lipoxin 4A has been reported to bind to AhR in guinea pig cells and to activate AhR-dependent gene expression in mouse cells (Schaldach et al. 1999). The synthetic female sex hormone ethisterone shares structural similarities with the estrogenic steroid hormone equilenin (Table S4). The latter has been shown to induce CYP1A1 mRNA in treated human hepatoma (HepG2) cell lines and to weakly displace ^3^H-BaP from AhR (Jinno et al. 2006). Another unique feature was the three flexible multi-fused ring structures with one hexa-chlorinated ring and the other rings with none or single hydrophilic groups, for example aldrin (309-00-2) (Cluster 27). Aldrin has been tested in the Tox21 initiative where it was, however, identified as inactive in the human HepG2-AhR-luciferase reporter gene assay (NCBI). A structurally similar compound to aldrin is hexachlorobenzene, which has been shown to induce AhR expression in the HepG2 cell line (de Tomaso Portaz et al. 2015). Aldrin and hexachlorobenzene are banned by the Stockholm Convention, but this is not the case for endosulfan alcohol (2157-19-9), which is also located in the same cluster (27). A number of benzothiazoles and thiazoles (Clusters 11 and 12, respectively) were identified as potential AhR binders. This included the earlier mentioned di(benzothiazol-2-yl) disulfide (120–78-5) (“Ligand-based methods” section) but also benzothiazoles that have an aliphatic ring structure. An example of such compounds is *N*-cyclohexyl-2-benzothiazolylsulfenamide (95-33-0) (Table S4). He et al. (2011) showed that several of such derivatives induce AhR-dependent luciferase reporter gene expression in recombinant mouse hepatoma cells. More examples and an extended analysis of some of the above-mentioned chemicals and clusters are given in the Supporting Information.

#### Evaluation of the virtual screening

Data from the Tox21 database (NCBI) on AhR-induced activity were available for 94 compounds (38 active and 56 inactive) out of the 429 compounds screened in this study. The 94 compounds covered a great part of the chemical domain set by our 429 compounds (Fig. S8). That is, the Tox21 chemicals are representative of the structural variation within the chemicals screened in the parallel steps. We calculated the accuracy and specificity of our screening method using active/inactive compounds and the results showed that the accuracy (0.61 and 0.63) and specificity (0.98 and 0.89) of the ligand-based methods were higher than the molecular docking method (0.53 and 0.54). The lower specificity of the molecular docking indicates that it more often generate false positives. The docking showed, however, higher sensitivity (0.53) than the ligand-based methods (0.05 and 0.24); it identified 20 of the 38 active compounds and 15 compounds of these were identified as potential AhR ligands solely by the docking (Dataset S8). These chemicals often included multiple unfused aromatic rings, nitrogen-containing five-membered rings, aliphatic chains as substituents or as a bridge between aromatic rings, and they are generally branched (e.g., 1-(4-chlorophenyl)-3-(3,4-dichlorophenyl)urea (101-20-2)). Notably, 14 of the 38 active compounds were never identified as potential AhR ligands by any of the parallel steps (Dataset S8) and these chemicals were often small, rigid aromatic compounds with few substituents. The virtual screening protocol is based on structural similarities to known AhR binders and estimated binding to AhR. However, AhR activation as used in this comparison may not always be dependent on binding (Denison and Nagy 2003; Nguyen and Bradfield 2007). Compounds that activate AhR, for instance, by cross-talking to other nuclear receptors, as suggested by Denison et al. (2011), may be hard to identify by our virtual screening protocol. An additional uncertainty is species-specific variation as the virtual screening protocol was based on data from rat, whereas the Tox21 data was derived using human cells.

### Potential AhR ligands

**Fig. 5:**
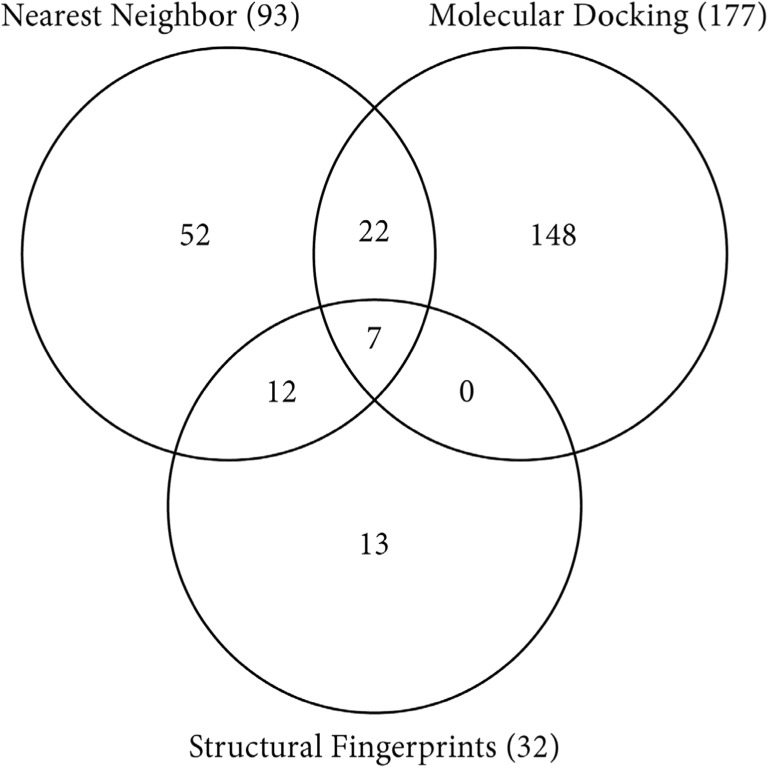
The number of potential AhR ligands identified by the three parallel virtual screening methods of nearest neighbor analysis based on chemical/physical properties, structural fingerprints, and molecular docking with an AhR homology model. Out of the 429 industrial chemicals identified in the initial filtering procedure that were used as input for the 3 screening methods, the numbers refer to the numbers of compounds identified by the 3 methods. A total of 41 compounds were identified as potential AhR ligands by at least 2 methods, and 7 compounds were identified by all 3 methods

**Fig. 6:**
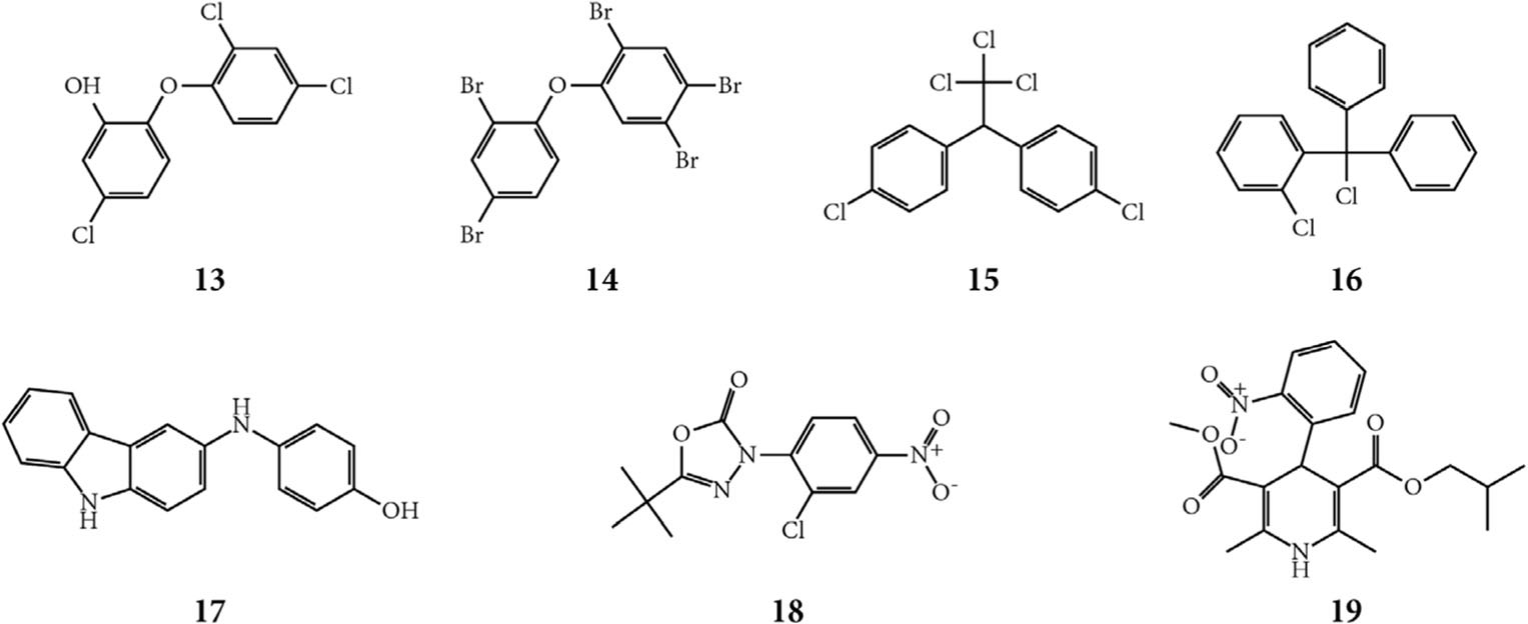
Chemical structures of the seven compounds identified as potential AhR ligands by all three parallel methods in the virtual screening protocol. Triclosan (**13**), 2,2′,4,4′,5-pentabromodiphenylether (BDE99) (**14**), p,p’-dichlorodiphenyltrichloroethane (pp’-DDT) (**15**), 2-chlorotritylchloride (**16**), 3-p-hydroxy-anilinocarbazole (**17**), 3-(2-chloro4-nitrophenyl)-5-(1,1-dimethylethyl)-1,3,4-oxadiazol-2(3H)-one (**18**), and nisoldipine (**19**)

The virtual screening protocol resulted in 41 compounds that were identified by at least 2 of the 3 parallel methods (Fig. 5, Table S5) among which 7 were identified by all 3 enrichment methods (Fig. 6). We used the Tox21 data set to compare the reported classification regarding AhR activity (active agonist, inactive), and also possible AhR antagonism (inactive, inconclusive antagonist, active antagonist), to our selection of 41 potential AhR ligands, hereafter called the consensus compounds. Eight of the consensus compounds had been tested in Tox21 (“Evaluation study of the virtual screening protocol” section) (NCBI). Four of these eight reported chemicals were classified as “active agonist,” one as “inconclusive agonist” (triclosan), and the remaining three as “inactive” (Dataset S8). However, two of the “inactive” chemicals have been reported in the literature to either suppress or activate AhR (de Oca et al. 2015; NCBI; Wojtowicz et al. 2011). Among the consensus compounds, 17 were halogenated, 16 contained fused rings, and 14 were atypical potential ligands. To our knowledge, AhR-related activation or suppression has been reported for 9 of the 41 chemicals in the scientific literature (Table S7). Together with the compiled data from the Tox21 database (NCBI) (Table S8), in total 13 compounds have been tested for AhR-mediated effects with a purity > 90%. That is, 28 of the consensus compounds are thus prime candidates for future AhR-related in vitro testing (Table S8).

The ligand-based enrichment steps (structural fingerprints and nearest neighbor analysis) jointly identified 12 compounds, and 22 compounds were jointly identified by the nearest neighbor and molecular docking enrichments (Fig. 5, Table S7). The structural fingerprints and molecular docking methods did not identify any compounds in common other than those that were already identified by all three enrichment methods. The consensus compounds included 11 chemicals registered in REACH,5 were identified as compounds being produced between 100 and 100,000 tons per year, and 6 were registered for intermediate usage (ECHA) (Table S6). Three of these 11 compounds, i.e., di(benzothiazol-2-yl) disulfide, triclosan, and bumetrizole, have been reported to activate or suppress AhR (Table S7).

The seven compounds that were identified by all three enrichment methods included structural analogues of both typical (five compounds) and atypical (two compounds) AhR binders and these were aromatic, mainly halogenated (five out of seven compounds), and relatively hydrophobic (Fig. 6). These compounds covered well-studied environmental contaminants, including the biocide triclosan, the brominated flame retardant 2,2′,4,4′,5-pentabromodiphenylether (BDE99), and the pesticide p,p’-dichlorodiphenyltrichloroethane (pp’-DDT). The DDT isomers pp’-DDT and o,p’-dichlorodiphenyltrichloroethane and their metabolites have been shown to suppress the activity of CYP1A1 in human placenta cells (Wojtowicz et al. 2011), but were identified as inactive in the Tox21 screening (NCBI), both regarding agonism and antagonism. BDE99 has been shown to be a partial AhR-agonist, and also an antagonist with an EC_50_ value of 13 μM (Hamers et al. 2006) and nisoldipine was identified as an agonist with a potency of 3.9 μM in the Tox21 program (NCBI). The AhR-related activity of triclosan has been studied by Ahn et al. (2008) who concluded that it is a weak agonist using an in vitro luciferase reporter gene assay based on rat hepatoma cells (note it was reported as “inconclusive agonist” in the Tox21 data base (NCBI)). To our knowledge, AhR-mediated effects have not been studied for the remaining three compounds. These include the pesticide 2-chlorotrityl chloride, the dye 3-*p*-hydroxyanilino-carbazole, and the herbicide precursor 3-(2-chloro-4-nitrophenyl)-5-(1,1-dimethylethyl)-1,3,4-oxadiazol-2(3H)-one (Gidwani et al. 2002; Pilgram 1979). 2-Chlorotrityl chloride shares structural similarities with typical AhR binders such as PAHs and PCBs, whereas the two latter compounds have structural similarities to the atypical binders FICZ and flutamide.

Among the 12 solely ligand-based hits, i.e., the compounds that were identified in both the nearest neighbor analysis and the structural fingerprints, 3 were halogenated and 3 included fused rings. Three of the 12 were atypical binders, including 2-amino-5-nitrobenzophenone (1775-95-7), 2-chloroethyl 3-nitro-*p*-toluate (59383-11-8), and 4-methoxy-3-nitro-*N*-phenylbenzamide (97-32-5). The following halogenated compounds were identified: 2,6-dichloro-*N*-phenylaniline (15307-93-4), 2-chloroethyl 3-nitro-*p*-toluate (59383-11-8), and 4-bromo-2-fluoro-1,1′-biphenyl (41604-19-7). Three of the hits resembled PAHs, including anthracene-9-carbaldehyde (642-31-9), 3-(9-anthryl) acrylaldehyde (38982-12-6), and 2,8-dimethylnaphtho(3,2,1-kl)xanthene (81-37-8) (Table S5). 2,6-Dichloro-*N*-phenylaniline is an analogue to the anti-inflammatory drug diclofenac and has been used to synthesize diclofenac analogues in an attempt to identify new anti-inflammatory drug candidates (Moser et al. 1990). Anthracene-9-carbaldehyde is a common intermediate in the production of dyes and pigments and has been shown to be metabolized mainly by CYP2B1 and CYP2C11 in rats and primarily by CYP3A (and to some extent by CYP2A6, CYP2B6, and CYP2C9) in human liver microsomes (Marini et al. 2003). Among these 12 compounds was terphenyl, which is generally a mixture of the 3 isomers *ortho*-, *meta*-, and *para*-terphenyl (where the *para* isomer is dominating in technical mixtures). Terphenyl has been used as a dye since the 1980s and more recently in new nanomaterials due to its optical properties (Fan et al. 2010; Liphardt and Luettke 1981).

The structure-based and the nearest neighbor enrichment processes identified 22 common compounds, and among these 9 were halogenated, 12 had fused ring systems, and 9 were atypical. Among the halogenated compounds was dicofol, a hydroxylated derivative of DDT (Ricking and Schwarzbauer 2012) that has a weak thyroidogenic effect (Ishihara et al. 2003). Moreover, these 22 compounds included many that have hydrogen acceptors in similar positions as the known atypical AhR binders ITE and FICZ. Examples of such compounds are bumetrizole (3896-11-05), di(benzothiazol-2-yl) disulfide (120-78-5), and 5-chloro-2-(2,4-dichlorophenoxy)aniline (56966-52-0). Bumetrizole is a benzotriazole used as an ultraviolet absorption stabilizer (UV326), and this compound has been shown to activate AhR-related pathways through the induction of *cyp1a1* and *AHR2* in zebra fish embryos (Fent et al. 2014). Benzothiazoles, such as di(benzothiazol-2-yl) disulfide, are used as vulcanization accelerators in rubber production (Wik and Dave 2009), and these compounds have been identified as a new class of AhR agonists in a recent study of 16 benzothiazoles (He et al. 2011). 5-Chloro-2-(2,4-dichlorophenoxy)aniline is an analogue of triclosan (a hydroxyl group is replaced by an amino group) and is an intermediate in the synthesis of other triclosan analogues, and it has significant potency against drug-resistant strains of the human malaria parasite *Plasmodium falciparum* (Anderson et al. 2013).

## Conclusions

We have developed a virtual screening protocol to identify potential AhR ligands among a set of industrial chemicals. Overall, relatively few compounds were identified as potential AhR ligands among the 6445 industrial chemicals studied here. This suggests that among industrial chemicals, only a small fraction is AhR ligands and that there are very few compounds that resemble the most well-studied AhR ligands. In total, only 41 industrial chemicals were identified by at least two of the three parallel steps. Among these 41 industrial chemicals, 4 compounds have been reported as AhR agonists and 1 compound as a possible AhR agonist in human in vitro assays. Moreover, according to the literature, another 6 compounds among the 41 have been identified to induce CYP1A1. The evaluation of the protocol using Tox21 data showed that the ligand-based methods had higher accuracy than the structure-based method (i.e., the molecular docking). In contrast, the molecular docking protocol identified more of the active compounds in the Tox21 database. The protocol is, however, not able to differentiate between AhR agonists and antagonists. The multivariate analysis of 214 known AhR modulators identified in the existing literature showed that most of these compounds belong to the typical chemical classes related to AhR activation. Of these 214 known modulators, approximately 40 compounds had more flexible/aliphatic, larger, and more complex structures (e.g., compounds with atom types other than combinations of carbon, hydrogen, halogens, and oxygen). The structure-based screening, which consisted of molecular docking using a rat AhR homology model, emphasized the versatile nature of AhR and indicated that AhR interacts with both non-aromatic polycyclic compounds and aromatic-substituted hydrocarbons. Among the 41 compounds identified as potential AhR ligands, 28 are prime candidates for further in vitro studies. In particular, we strongly suggest detailed studies on 2-chlorotrityl chloride, 3-p-hydroxyanilino-carbazole, and 3-(2-chloro-4-nitrophenyl)-5-(1,1-dimethylethyl)-1,3,4-oxadiazol-2(3H)-one. In addition, we suggest further screening of AhR-induced activities for the 429 industrial chemicals to enable a better validation of the developed virtual screening protocol including in particular chemicals that resemble atypical AhR ligands.

## Electronic supplementary material

ESM 1 Additional information on the selection of the 66 known AhR binders, the PCA of the 214 known AhR modulators, the comparisons of EROD and CALUX data, the histograms of ΔG_bind_-values from the rescoring, the PCA of the 429 industrial chemicals and AhR binders after the initial screening step, representative structures from the parallel virtual screening steps, and the structures of the final selection of the 41 potential AhR ligands. The supporting material also includes a file of datasets, including the median relative effect potencies (REPs) for the AhR modulators, the Simplified Molecular Input Line Entry Specification (SMILES) of the AhR modulators, the AhR binders, the subsets of industrial chemicals after the different steps in the virtual screening protocol, and of the final list of potential AhR ligands. Supporting information can be sent on request by contacting the corresponding author. (PDF 1916 kb)

## Abbreviations

2378-TCDD: 2,3,7,8-tetrachlorodibenzo-*p*-dioxin
23479-PeCDD: 2,3,4,7,9-pentachlorodibenzo-*p*-dioxin
124678-HxCDD: 1,2,4,6,7,8-hexachlorodibenzo-*p*-dioxin
AhR: aryl hydrocarbon receptor
BaP: benzo-a-pyrene
BDE99: 2,2′,4,4′,5-pentabromodiphenylether
CAS: chemical abstracts service;
DNF: dinaphtho[1,2-b;1′2’-d]furan
DR-CALUX: dioxin responsive chemically activated luciferase expression;
DModX: distance to the model
ED: Euclidean distance
EROD: ethoxyresorufin-*O*-deethylase
FICZ: 6-formylindolo[3,2-b]carbazole
H/LPVC: high and low production volume chemicals
ITE: 2-(1’H-indole-3′-carbonyl)-thiazole-4-carboxylic acid ester
LBD: ligand binding domain
MACCS: molecular access system
MM-GBSA: molecular mechanics generalized born/surface area
PC: principal component
PCA: principal component analysis
HOMO: highest occupied molecular orbital
LUMO: lowest unoccupied molecular orbital
PCB: polychlorinated biphenyl
PCB126: 3,3′,4,4′,5-pentachlorobiphenyl
PCB157: 2,3,3′,4,4′,5′-hexachlorobiphenyl
PCB77: 3,3′,4,4′-tetrachlorobiphenyl
PCDD/Fs: polychlorinated dibenzo-*p*-dioxins and dibenzofurans
pp’-DDT: p,p’-dichlorodiphenyltrichloroethane
R^2^X: explained variation in each PC
REACH: registration, evaluation, authorization and restriction of chemicals
REP: relative effect potency
SMILES: simplified molecular-input line-entry system
TC: Tanimoto coefficient
