# Supplementary material for: Identification of potential aryl hydrocarbon receptor ligands by virtual screening of industrial chemicals
Source: Environ Sci Pollut Res Int. 2017 Nov 10;25(3):2436–49. doi: 10.1007/s11356-017-0437-9 (PMC5773624; doi:10.1007/s11356-017-0437-9)
Supplement: Supplementary file 1 — Additional information on the selection of the 66 known AhR binders, the PCA of the 214 known AhR modulators, the comparisons of EROD and CALUX data, the histograms of ΔGbind-values from the rescoring, the PCA of the 429 industrial chemicals and AhR binders after the initial screening step, representative structures from the parallel virtual screening steps, and the structures of the final selection of the 41 potential AhR ligands. The supporting material also includes a file of datasets, including the median relative effect potencies (REPs) for the AhR modulators, the Simplified Molecular Input Line Entry Specification (SMILES) of the AhR modulators, the AhR binders, the subsets of industrial chemicals after the different steps in the virtual screening protocol, and of the final list of potential AhR ligands. Supporting information can be sent on request by contacting the corresponding author. (PDF 1916 kb) [file 11356_2017_437_MOESM1_ESM.pdf]

Supporting Information of

Identification of potential aryl hydrocarbon receptor ligands by virtual screening of industrial chemicals

Malin Larsson, Domenico Fraccalvieri, C. David Andersson, Laura Bonati, Anna Linusson, and Patrik L. Andersson

Contents

In vitro data evaluation process and relative effect potency (REP) calculations 3

Additional information on the virtual screening protocol 4

Additional principal component analysis of the 214 AhR modulators 6

Additional analysis of the chemicals from the structural-based method 6

Figures 7

Figure S1a. Relative effect potencies (REPs) of the compounds measured with both ethoxyresorufin-O-deethylase (EROD) and luciferase reporter gene assays (dioxin-responsive Chemical Activated LUciferase gene eXpression, DR-CALUX), based on the effective concentration equal to 20% of the maximum induction (EC_{20}) (Behnisch et al. 2003)................................ 8

Figure S1b. Relative effect potencies of the compounds measured with both ethoxyresorufin-O-deethylase (EROD) and luciferase reporter gene assays (dioxin-responsive Chemical Activated LUciferase gene eXpression, DR-CALUX), based on the effective concentration equal to 50% of the maximum induction (EC_{50}) (Behnisch et al. 2003)................................ 8

Figure S2a. Relative effect potency values based on the effective concentration equal to 20 and 50% of the maximum induction (EC_{20}, EC_{50}) using the dioxin-responsive Chemical Activated LUciferase gene eXpression (DR-CALUX) assay (Behnisch et al. 2003)................................ 9

Figure S2b. Relative effect potency values based on the effective concentration equal to 20 and 50% of the maximum induction (EC_{20}, EC_{50}) using ethoxyresorufin-O-deethylase (EROD) assay (Behnisch et al. 2003). 9

Figure S3a. The score plot of the first, second and third principal component for the PCA of all 214 modulators. 10

Figure S3b. The loading plot of the first, second and third principal component for the principal component analysis of all 214 AhR modulators 10

Figure S4a. The score plot of the second and fifth principal component for the principal component analysis of the 214 AhR modulators obtained in the literature study................................ 11

Figure S4b. The loading plot of the second and fifth principal component for the principal component analysis of the 214 AhR modulators obtained in the literature study................................ 11

Figure S5. Histograms of the lowest ligand binding energies (ΔG values) of each docked compound using the up to ten top-poses (from the molecular docking) followed by rescoring with the molecular mechanics generalized Born/surface area (MM-GBSA) protocol for the AhR binders

(upper) and the remaining 429 industrial chemicals. Only 392 were able to be docked successfully and therefore in the end obtain ΔG-values. 12

Figure S6a. The score plot of the first and second (t_1 and t_2) principal component of the principal component analysis of the 429 industrial chemicals (yellow and blue) and the 214 AhR modulators (including the 66 AhR binders) (grey, green and turquoise), explaining 38% and 18%, respectively, of the variation in the data 13

Figure S6b. The loading plot of the first and second (p_1 and p_2) principal components of the principal component analysis of the 429 industrial chemicals and the 214AhR modulators (including the 66 AhR binders), explaining 38% and 18%, respectively, of the variation in the data. 14

Figure S7. Clusters of compounds as a result of a hierarchical clustering where each frame represents a cluster, the cluster numbers are given in the upper left corner, the number of cluster members in the right corner with the number of known binders within brackets, and where the molecule was the centre of each cluster. 15

Figure S8a. The score plot of the principal component analysis of the 429 industrial chemicals screened in the parallel steps and the 66 AhR binders. The AhR binders are here depicted as green dots, the compounds tested within Tox21 are depicted as red dots, and the remaining chemicals as yellow dots. The variation in the PCs is 36 % and 18 % respectively. 16

Figure S8b. The loading plot of the first and second (p_1 and p_2) principal components of the principal component analysis of the 429 industrial chemicals and the 66 AhR binders, explaining 36% and 18%, respectively, of the variation in the data 17

Tables 18

Table S1. The 3D descriptors used in the principal component analysis of the 214 AhR modulators. The following descriptors depend on the structure connectivity and conformation (dimensions are measured in Ångstrom) (MOE 2012.10). 18

Table S2. Characteristics of the clusters found in the principal component analysis (PCA) of the 214 AhR modulators. 19

Table S3. Structures of the industrial chemicals discussed and compared with data from the literature in Section 3.2.2 in the main text. 20

Table S4. Structures of the industrial chemicals discussed and compared with data from the literature in Section 3.2.3 in the main text. 22

Table S5. The structures of the 41 industrial chemicals that were identified as potential AhR ligands. 24

Table S6. Registration information of the compounds (among the 41 jointly identified potential AhR ligands) that are registered in REACH [2015-05-28]. 28

Table S7. AhR-related response data for compounds among the 41 jointly identified potential AhR ligands found by search in SciFinder [2016-03-16]. 28

Table S8. Overview of tested AhR activity based on ToxCast and PubChem Bioassay databases within Tox21 for the 41 jointly identified potential AhR ligands. 29

Table S9. Comparisons of 94 compounds tested in the PubChem Bioassay and the enrichments from the parallel steps 30

References 30

In vitro data evaluation process and relative effect potency (REP) calculations

The search of compounds having shown aryl hydrocarbon receptor (AhR) mediated effects resulted in 214 compounds (Dataset S1), i.e. the AhR modulators. Out of these AhR modulators, 78 were measured in both ethoxyresorufin-O-deethylase (EROD) and luciferase reporter gene assays (dioxin-responsive Chemical Activated LUciferase gene eXpression, DR-CALUX) by a single research group (Behnisch et al. 2003). In that study, they calculated the relative effect potencies (REPs), i.e. the ratio between the EC-values of 2,3,7,8-tetrachlorodibenzo-p-dioxin (2378-TCDD) and the tested compound. 2,3,7,8-TCDD is usually used as reference when comparing potencies for AhR mediated responses, and REPs are used in the risk assessment of dioxins and dioxin-like compounds (van den Berg et al 2006). Comparing their *in vitro* relative effects potency(REP) measurements for these 78 compounds showed high correlation, both regarding EC_{20} and EC_{50} values (Figure S1) and when correlating EC_{20} data to EC_{50} data (Figure S2). That is, this showed the close relation in values between the two methods. Furthermore, these 78 compounds had EC_{20} and EC_{50} in the order of magnitude 10 pM to roughly 0.1 uM, i.e. a difference in REP value of 100 000 for the least active compound compared to 2378-TCDD (Behnisch et al. 2003).

For the investigated *in vitro* studies from the literature, REPs were calculated compared to the corresponding measurement of 2378-TCDD in the same study. Exceptions were the following; in the case of PCB 126 being measured instead as 2378-TCDD, the REP was made relative to PCB 126, and then extrapolated by a factor 10 to be comparable with REPs based on 2378-TCDD. The extrapolation was based on the fact that the WHO established risk assessment factors, i.e. toxic equivalency factors (TEFs) of 2378-TCDD and PCB 126 differ by one order of magnitude (Behnisch et al. 2003). In many cases, several articles had measured the same compound, i.e. multiple values were obtained. Due to that, the median REP was used as final result for the compound's potency. The median REPs were used as a rough measurement to find patterns and correlations in the PCA based on calculated physico-chemical properties (Figure S3, S4), that is, only for visual inspection. In addition, if 2378-TCDD was not tested for a compound, but a lower concentration (e.g. 1-10 nM) gave a significant increase in AhR-mediated effect or the compound was compared to another known AhR agonist (here, beta-naptho-flavone, BNF) (Casado et al. 2006), the compound was added but its REP value put as "Missing". This was also applied in cases where 2378-TCDD had been tested in the same study, and dose-response curves were reported but no actual EC-values for both compounds. This was the case of, for instance, the known AhR ligands 3-methyl cholanthrene (3-MC) and BNF (Denison et al. 2003, Garrison et al. 1996). Since the reported established K_d-value of 3-MC and K_i-value of BNF were 0.27 nM and 2.0 nM, respectively, the compounds were included in the data set of 66 AhR binders that induced AhR related activity as well as in the PCA of the 214 AhR modulators. In Dataset S1, REP values of 3-MC and BNF were set as missing (---), but the compounds seem to be approximately 1000 000 times less potent than 2378-TCDD, i.e. REP of $1*10^{-6}$, according to the dose-response curves provided by Garrison et al. In the literature search, the compound lipoxin 4A was encountered, which dramatically differs in structure compared to typical AhR ligands, such as 2378-TCDD (Schaldach et al. 1999). The compound was tested for AhR activity *in vitro*, and produced a concentration-dependent response in a dioxin-responsive element (DRE)-driven chloramphenicol acyltransferase (CAT) reporter gene construct. Even though it was not a luciferase reporter gene assay, it was decided interesting enough to include in the analysis of

AhR modulators. Furthermore, the compound also showed to increase the levels of cyp1a1 mRNA and to competitively bind to the AhR (half maximum inhibition, IC_{50}, = 0.1 uM) in experiments with guinea pig cells.

Additional information on the virtual screening protocol

The virtual screening protocol consisted of six steps (see Section 3.2 in the main text): pretreatment of the industrial chemicals (**1**), characterization based on physico-chemical properties and multivariate chemical space (**2**), three parallel steps (**3-5**) and a concluding analysis for the outcome of the parallel steps (**6**). Below follows additional information on the settings for some of the steps and the decision-making regarding cut-offs. The used statistical programs, methods and concepts are further described in the main text.

General data settings and procedures for the principal component analyses

Prior to all principal component analyses (PCAs), i.e. step 2, 2' (explained in next section) and 3, the descriptor data was scaled to unite variance. Significant principal components were defined as those having eigenvalues above 2, level of explained variation (R^2X) above 4%, combined with the interpretability of the PC. The PCAs in step 2 and 2' had four significant PCs, and the one in step 3 had five significant PCs.

Step 2: Physico-chemical properties

In step 2, the Hoteling T^2 values were used to detect and exclude outliers to the model (T^2crit(95%)= 10,5681), and used as the actual filter. The remaining compounds' Distance to Model (DModX)-values were though studied - they ranged between 0-7, most of them below 5 in values - but it was concluded that the ones having high DModX values still showed high similarity in structure to the 66 AhR binders, and that it would be misfortunate to exclude them this early on in the process.

An additional step, step 2', was added due to five AhR binders being located outside the initial T^2-Hoteling range in the PCA of the 66 AhR binders (Section 3.2.1); those compounds whose neighbors most likely were not fully accounted for in step 2. From the PCA of these five AhR binders (together with the 6,445 industrial chemicals), Euclidean distances were calculated and the cut-off was set to 1.5; higher distances did not share the same number of rings and/or similar functional groups in the right positions as the AhR binders. The descriptors for the PCAs in step 2' was, besides scaled, also log-transformed (using the auto-transform option in SIMCA 13.0) to normalize their distributions and to minimize the influence of extreme values originating from the industrial chemicals (Rannar and Andersson 2010).

Step 3: Structural fingerprints

The structural similarities between industrial chemicals and the 66 AhR binders were accounted for by calculating Tanimoto coefficients (TCs). A cut-off of 0.60 was chosen based on studying the output (and related structures) from a selection of the known AhR binders. For instance, a very structurally alike compound to one of the AhR binders (FICZ) was found having the value of 0.61; it only lacked one single bond to have the exact structural framework as FICZ. A TC of 0.50, on the other hand, showed to give compounds with very low resemblance to the tested known AhR binder, when visually inspecting the outcome (data not shown).

Step 4 : Nearest neighbor analysis based on chemical properties and chemical space

The cut-off in Euclidean distances to locate nearest neighbors was set by visually study the structures having the lowest distances and increasing it gradually. The PCA of the resulting industrial chemicals yielded five significant principal components (PCs) explaining 36%, 18%, 11%, 8%, and 5 % of the overall variation in the data, respectively. The first PC describes size and the second hydrophobicity (Figure S8b), the latter both regarding surface characteristics and octanol-water partition coefficient. Furthermore, the third PC describes aromaticity normalized to the total number of bonds in the structure, the fourth shape, degree of halogenation and number of rings regarding highly aromatic compounds, and the fifth describes the number of rings in relation to the compound's flexibility. The DModX values also show that the compounds are well fitted to the PCA model; the values were between 0 and 3, but all but five were below 2.

Step 5: Molecular docking

In the molecular docking, the 3D-optimized structures from step 2 and 2' were docked together with the 66 known AhR binders. Then these structures were rescored to estimate the energy of binding (ΔG_{bind}) between the chemicals and AhR, using the MM-GBSA method to account for interaction energies and desolvation effects occurring upon complex formation. The visual analysis of the binders' top-poses showed that all ligands were present and highly clustered to the same space of the pocket. Also the analysis showed that there was a trend in ΔG_{bind} -values rendering higher values for less potent congeners according to their IC_{50} values. For instance, PCDD/Fs with less than four lateral chlorines in the structures, achieved higher ΔG_{bind} than those with four lateral chlorines. Also PAHs received higher ΔG_{bind} than the PCDD/Fs with four lateral chlorines, which is consistent with the trend between PAHs and PCDD/Fs according to their IC_{50} values. All together this was taken as a proof of validity for the docking and rescoring procedure.

For each docked chemical, the pose with the lowest ΔG_{bind}, i.e. the strongest binder, was extracted for further analysis. Figure S5 shows the distributions of ΔG_{bind} -values from the industrial chemical data set and the AhR binders. The distributions from the two sets were fairly normal distributed and the distribution for the industrial chemicals was shifted to higher ΔG_{bind} as compared to the AhR binders. That is, the distributions showed that the known AhR binders were more probable to bind than the total collection of industrial chemicals. We estimated a cut-off based on the ΔG_{bind} values of the 65 known AhR binders (one failed in the docking procedure) which had an average ΔG_{bind} of −112.5 kcal/mol. The industrial chemicals that had a ΔG_{bind} within one standard deviation of the average ΔG_{bind} (−99.3 kcal/mol) of the 65 known binders were considered more likely to be potential AhR binders than those with higher ΔG_{bind}. This selection became the final enrichment from the molecular docking. Within this set of industrial chemicals no further ranking was made since that kind of accuracy was concluded not to exist. This conclusion was based on the fact that 2378-TCDD, the compound known to be the most potent AhR binder, only achieved a ΔG_{bind} slightly above average among the AhR binders (-106 kcal/mol). This indicated that the recorded ΔG_{bind} -values did not represent the exact ranking for each individual binder, and hence we could not expect the ranking to be more exact for the industrial chemicals.

Additional principal component analysis of the 214 AhR modulators

The first three PCs of the 214 AhR modulators showed four clusters: polycyclic aromatic hydrocarbons (PAHs), halogenated aromatics, natural products and flexible endogenous compounds (Figure S3). Pharmaceuticals and other small and less flexible/more rigid aromatic compounds (among the endogenous substances) were located at the center where all four clusters meet. The clusters were analyzed by the descriptors presented in the loading plot (Figure S3b) and by creating contribution plots (SIMCA 13.0) from marking the clusters, two at the time, in the PCA. Table 2 includes some of the characteristics of the located clusters and differences between them (regarding PC 1-3). For instance, a difference between halogenated aromatics and PAHs was that the PAHs had higher HOMO energy than the halogenated aromatics, and the latter had higher GAP than the PAHs (Table 1). McKinney et al. showed that a compound with greater ability to accept electrons through charge-transfer interaction could bind to the AhR with greater affinity (McKinney and Pedersen 1986), and earlier studies have shown that GAP and HOMO are important variables for QSAR modeling of polychlorinated dibenzo-*p*-dioxins and dibenzofurans (PCDD/Fs) and polychlorinated biphenyls (PCBs) (Larsson et al 2013). The score plot of the second and fifth PC managed to roughly capture the toxicological trend given by the median REPs (Figure S4), at least for the halogenated aromatics and PAHs. This indicates that shape descriptors and measures of density in high extent were able to gradually discriminate between less potent halogenated aromatic compounds.

Additional analysis of the chemicals from the structural-based method

A unique feature captured by the molecular docking was the steroid ring structures. A class with steroid ring structure, the ginsenosides, recently demonstrated to include naturally occurring weak AhR agonists and antagonists based on rat, mouse, guinea pig luciferase reporter gene assays combined with a guinea pig competitive binding assay (Hu et al. 2013). As the ginsenosides, a handful of the 177 compounds had hydrophilic groups or side-chains connected to rings at both ends. One example is cholic acid (81-25-4), which is discussed in the main text. The other mentioned steroid, ethisterone (434-03-7) share similarities with another known AhR binder and inducer equilenin (Jinno et al. 2006). Both compounds have a keto-group and a hydroxyl-group at lateral positions in the structures and a methyl-group on the same ring as the keto-group (Table S4). This implies that they could interact with AhR in a similar fashion. Besides steroids, also aliphatic 3-fused ring structures were promoted to bind to the AhR in the molecular docking and rescoring. An example of this is methyl tetrahydroabietate (MTHB, 19941-28-7) (cluster 6) which has three fused carbon rings and lateral substituents. To our knowledge, structures like MTHB have not earlier been tested for AhR-mediated effects. Compared to cholic acid, the carboxylic acid feature is replaced by an ester functional group and the hydroxyl group by an iso-propyl group. There were many other compounds that we believe did not resemble any known AhR ligands. Among these were, for instance, compounds with numerous hydrogen-acceptors and one or two aromatic rings (cluster 28 and 29). One example is the tris(oxiranylmethyl) benzene-1,2,4-tricarboxylate (7237-83-4), a benzene derivative which is a triple ester with epoxide groups (cluster 28). The drug ambroxol (18683-91-5), a common in cough syrup and in other treatment of respiratory diseases, is an example of another combination of structural features (cluster 8). As endosulfan alcohol (2157-19-9) (Table S4), it has one halogenated and one hydrophilic, i.e. hydroxylated, lateral end of the structure. However, it also contains nitrogen atoms as bridging atoms in the carbon chains or as amine group. The same three features in the same

order, i.e. halogens, nitrogen, hydrophilic groups, in a two ring structure are encountered in another drug, vinclozolin. Vinclozolin has shown to modulate hepatic cytochrome P450 isoforms in pregnant rats (de Oca et al. 2015). In cluster 12, three of the four benzothiazoles with an aliphatic ring structure had been tested by He et al. : N,N-dicyclohexylbenzothiazole-2-sulphenamide (4979-32-2), 2-(morpholinodithio)benzothiazole (95-32-9) and N-cyclohexyl-2-benzothiazolylsulfenamide (95-33-0) (Table S4). Although the high structural similarities the outcome of the *in vitro* assay were quite different. N-cyclohexyl-2-benzothiazolylsulfenamide was among the most potent tested derivatives, together with di(benzothiazol-2-yl) disulphide. Both compounds induced close to 70% luciferase activity at 10 uM concentration of the compounds, when compared to the activity of 2378-TCDD at 1 nM. However, N,N-dicyclohexylbenzothiazole-2-sulphenamide only induced close to 10 % luciferase activity in the same study, and 2-(morpholinodithio)benzothiazole showed no activity. The fourth benzothiazole with an aliphatic ring structure was 2-(morpholinothio)benzothiazole (102-77-2) which is used as a vulcanization accelerator in rubber industry (Lewis and Lewis 2016). This compound has structural similarities with the inactive 2-(morpholinodithio)benzothiazole (95-32-9), but also with the active 2-(morpholino-dithio)benzothiazole. The only difference in the structures is the sulfur, double sulfur or no bridging atom between the morpholine and the benzothiazole ring. Hence, it is hard to, on beforehand, predict the potency of 2-(morpholinothio)benzothiazole in a similar AhR mediated *in vitro* study.

Figures

The data for Figures S1 and S2 belonged to the following chemical classes: PAHs, polybrominated biphenyls (PBBs), polybrominated and chlorinated dibenzo-p-dioxins (PBCDDs), polybrominated and chlorinated dibenzofurans (PBCDFs), polybrominated dibenzo-p-dioxins (PBDDs), polybrominated diphenyl ethers (PBDEs), polybrominated dibenzofurans (PBDFs), polychlorinated biphenyls (PCBs), polychlorinated dibenzo-p-dioxins (PCDDs), polychlorinated dibenzofurans (PCDFs), polychlorinated napthalenes (PCNs), fluorinated or iodized PCDD/Fs (PXCDD/Fs), methylated PCDD/Fs (PYCDD/Fs), and various compounds like tetrabromobisphenol A (TBBP-A).

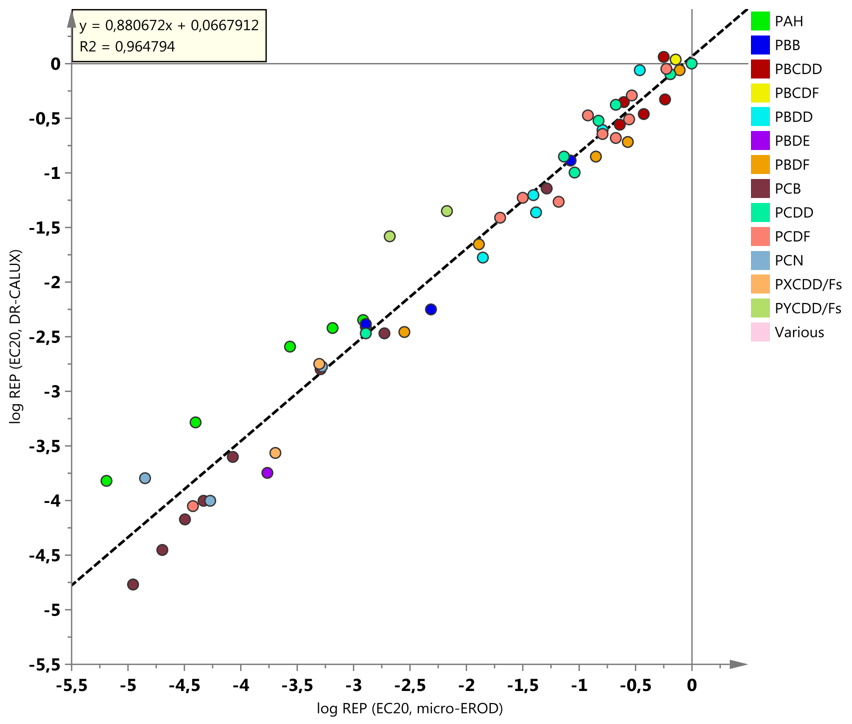

Figure S1a. Relative effect potencies (REPs) of the compounds measured with both ethoxyresorufin-O-deethylase (EROD) and luciferase reporter gene assays (dioxin-responsive Chemical Activated LUciferase gene eXpression, DR-CALUX), based on the effective concentration equal to 20% of the maximum induction (EC_{20}) (Behnisch et al. 2003).

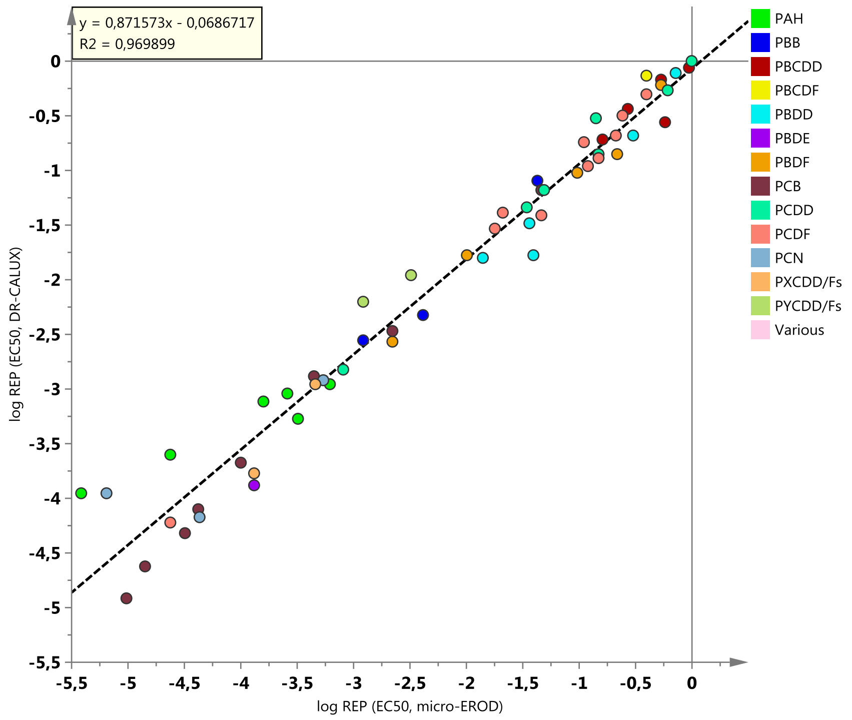

Figure S1b. Relative effect potencies of the compounds measured with both ethoxyresorufin-O-deethylase (EROD) and luciferase reporter gene assays (dioxin-responsive Chemical Activated LUciferase gene eXpression, DR-CALUX), based on the effective concentration equal to 50% of the maximum induction (EC_{50}) (Behnisch et al. 2003).

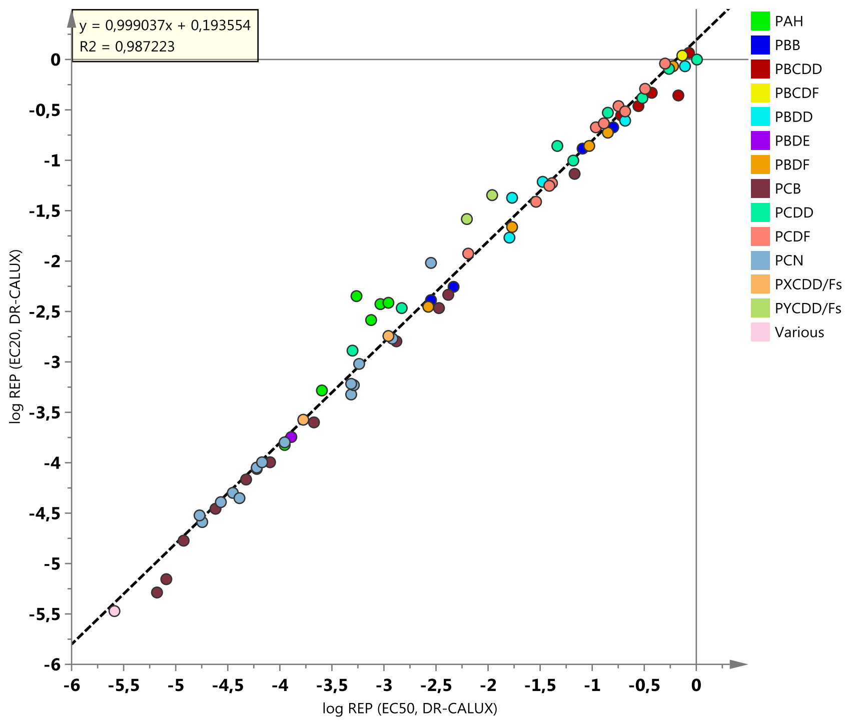

Figure S2a. Relative effect potency values based on the effective concentration equal to 20 and 50% of the maximum induction (EC_{20}, EC_{50}) using the dioxin-responsive Chemical Activated LUciferase gene eXpression (DR-CALUX) assay (Behnisch et al. 2003).

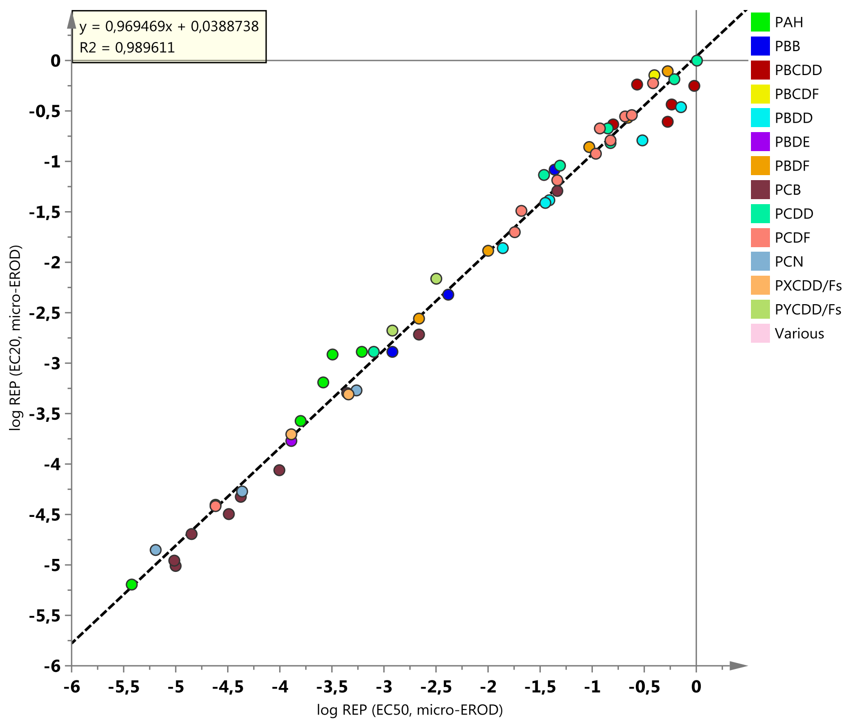

Figure S2b. Relative effect potency values based on the effective concentration equal to 20 and 50% of the maximum induction (EC_{20}, EC_{50}) using ethoxyresorufin-O-deethylase (EROD) assay (Behnisch et al. 2003).

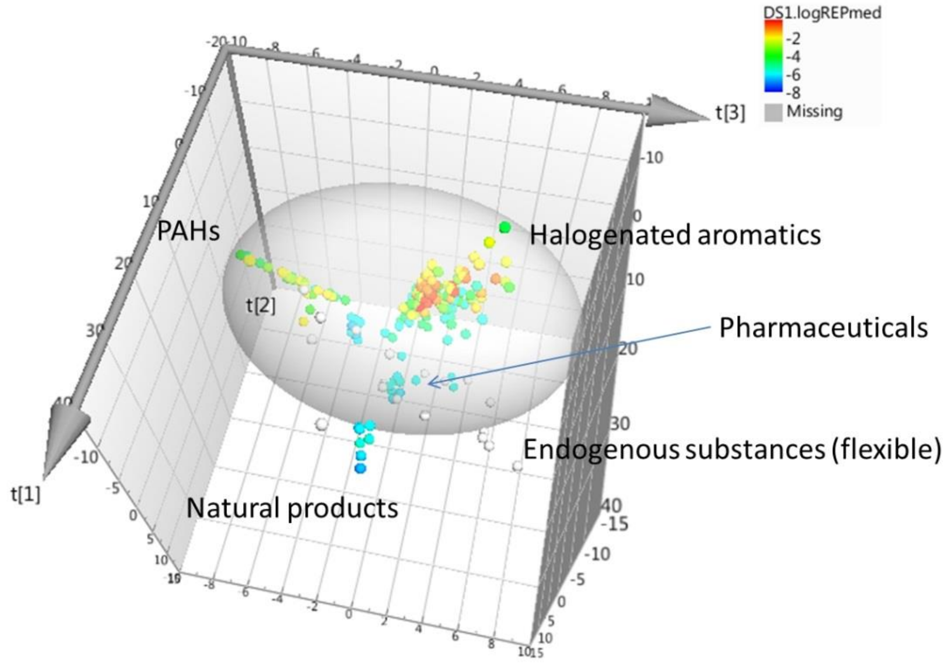

Figure S3a. The score plot of the first, second and third principal component for the PCA of all 214 modulators.

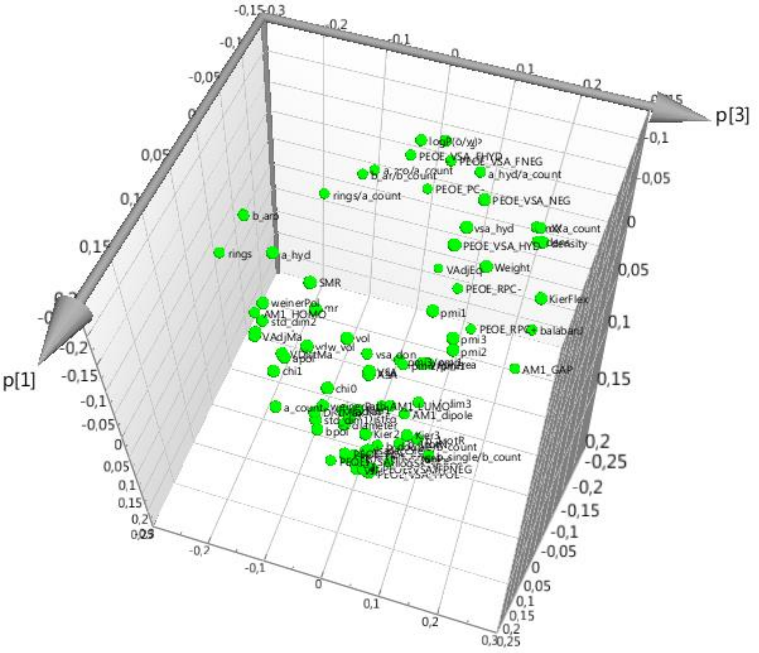

Figure S3b. The loading plot of the first, second and third principal component for the principal component analysis of all 214 AhR modulators.

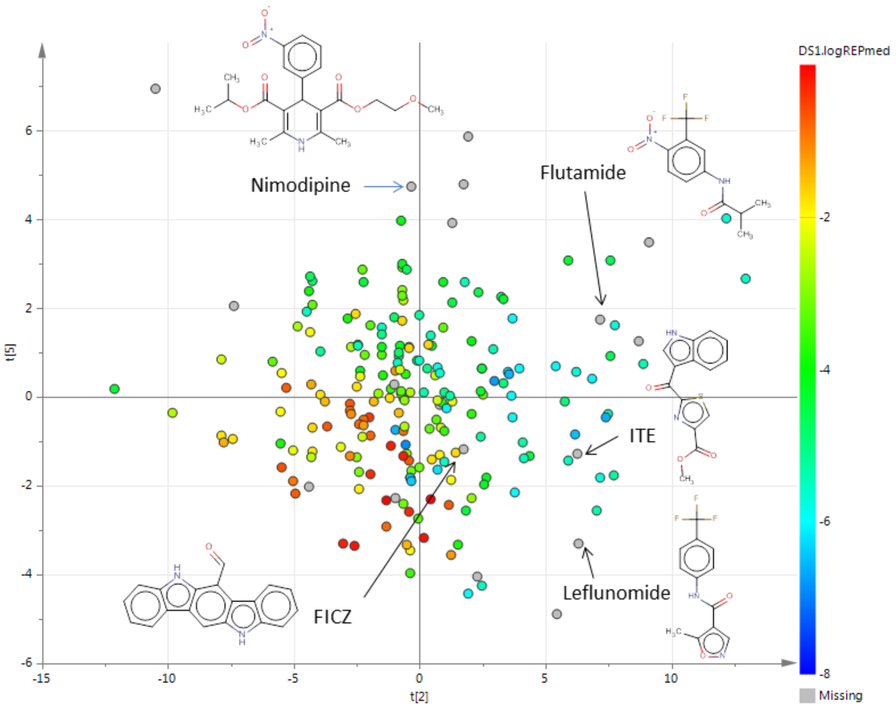

Figure S4a. The score plot of the second and fifth principal component for the principal component analysis of the 214 AhR modulators obtained in the literature study.

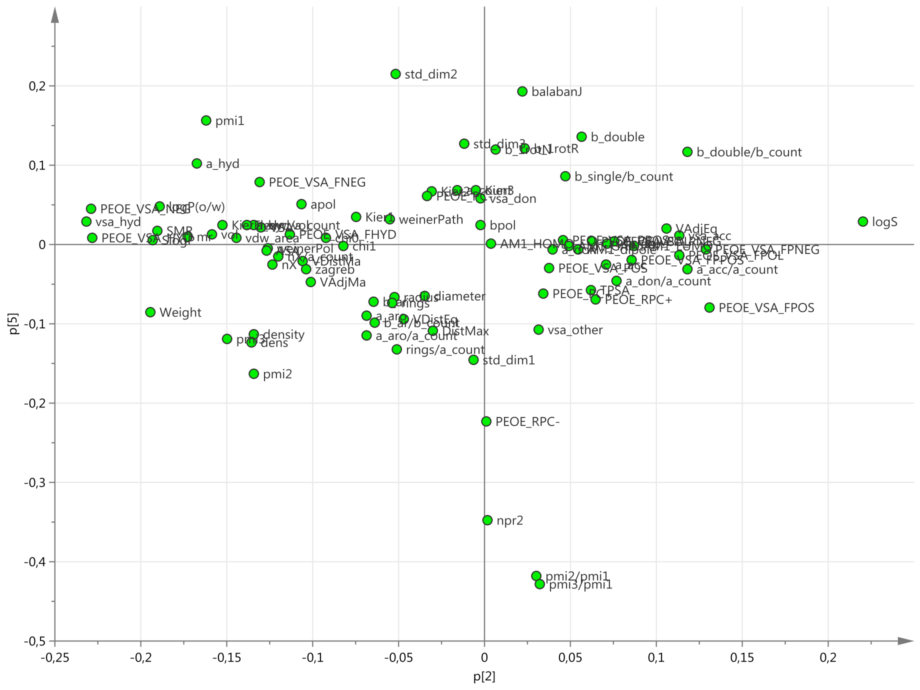

Figure S4b. The loading plot of the second and fifth principal component for the principal component analysis of the 214 AhR modulators obtained in the literature study.

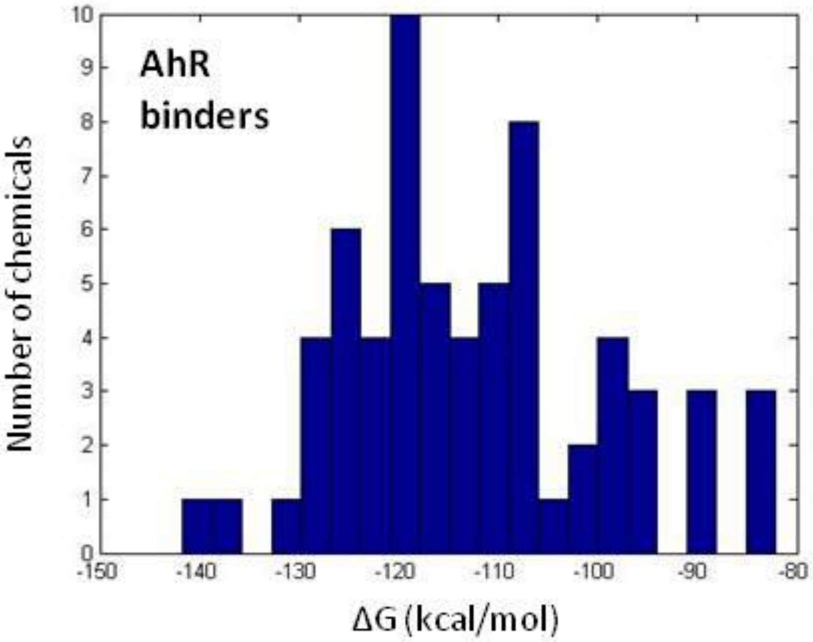

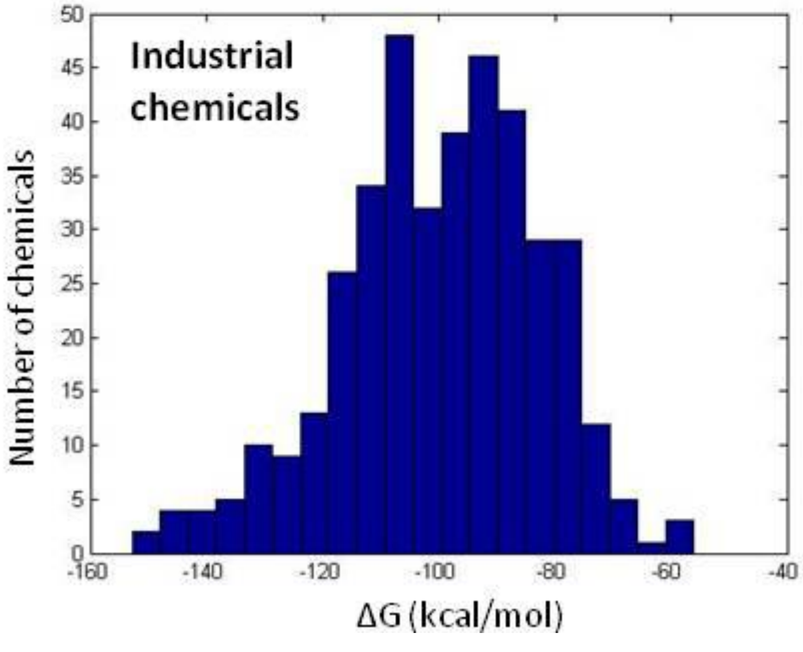

Figure S5. Histograms of the lowest ligand binding energies (ΔG values) of each docked compound using the up to ten top-poses (from the molecular docking) followed by rescoring with the molecular mechanics generalized Born/surface area (MM-GBSA) protocol for the AhR binders (upper) and the remaining 429 industrial chemicals. Only 392 were able to be docked successfully and therefore in the end obtain ΔG-values.

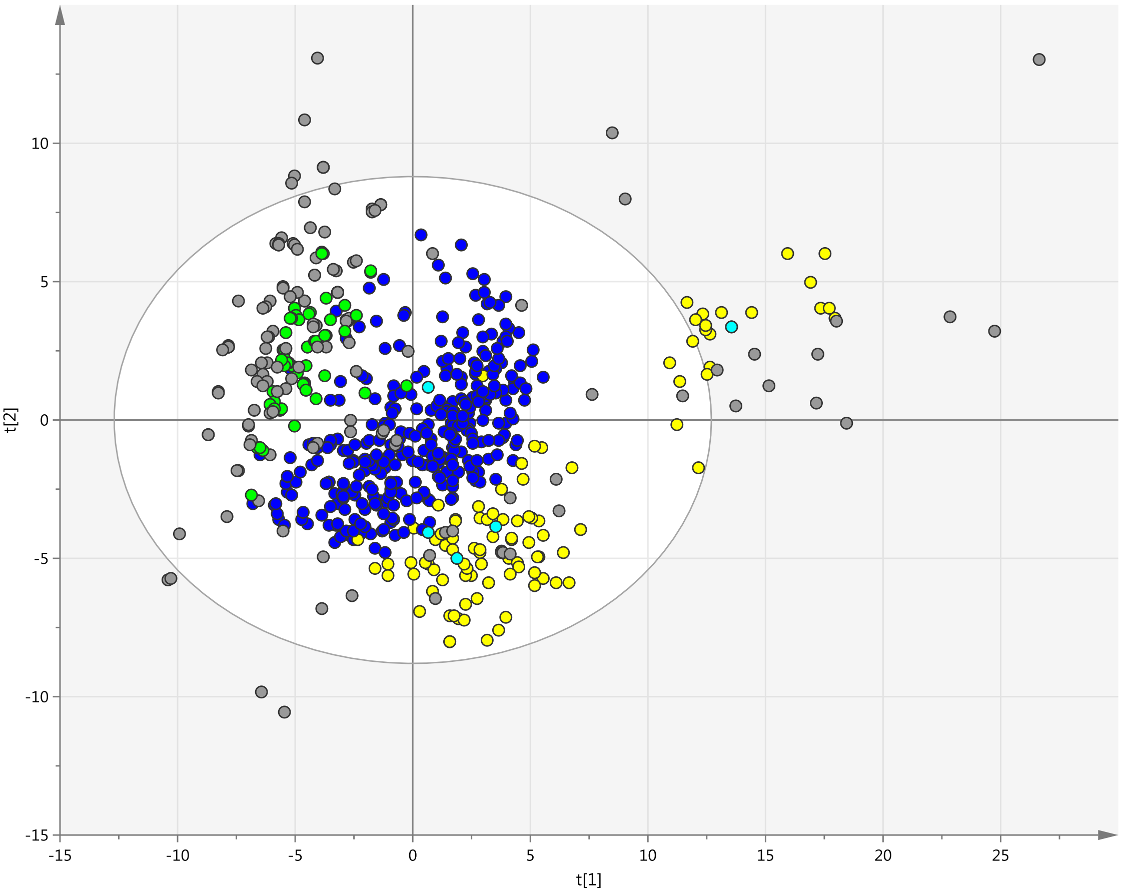

Figure S6a. The score plot of the first and second (t_1 and t_2) principal component of the principal component analysis of the 429 industrial chemicals (yellow and blue) and the 214 AhR modulators (including the 66 AhR binders) (grey, green and turquoise), explaining 38% and 18%, respectively, of the variation in the data. The five structurally diverse AhR binders (turquoise) (according to T2 hoteling 95%), i.e. atypical binders, the remaining AhR binders depicted as green dots (typical binders), the results from the T2 hoteling filter (step2) as blue dots, the additional industrial chemicals added from Euclidean distances from the five atypical AhR binders as yellow dots, and the remaining 148 AhR modulators as grey dots.

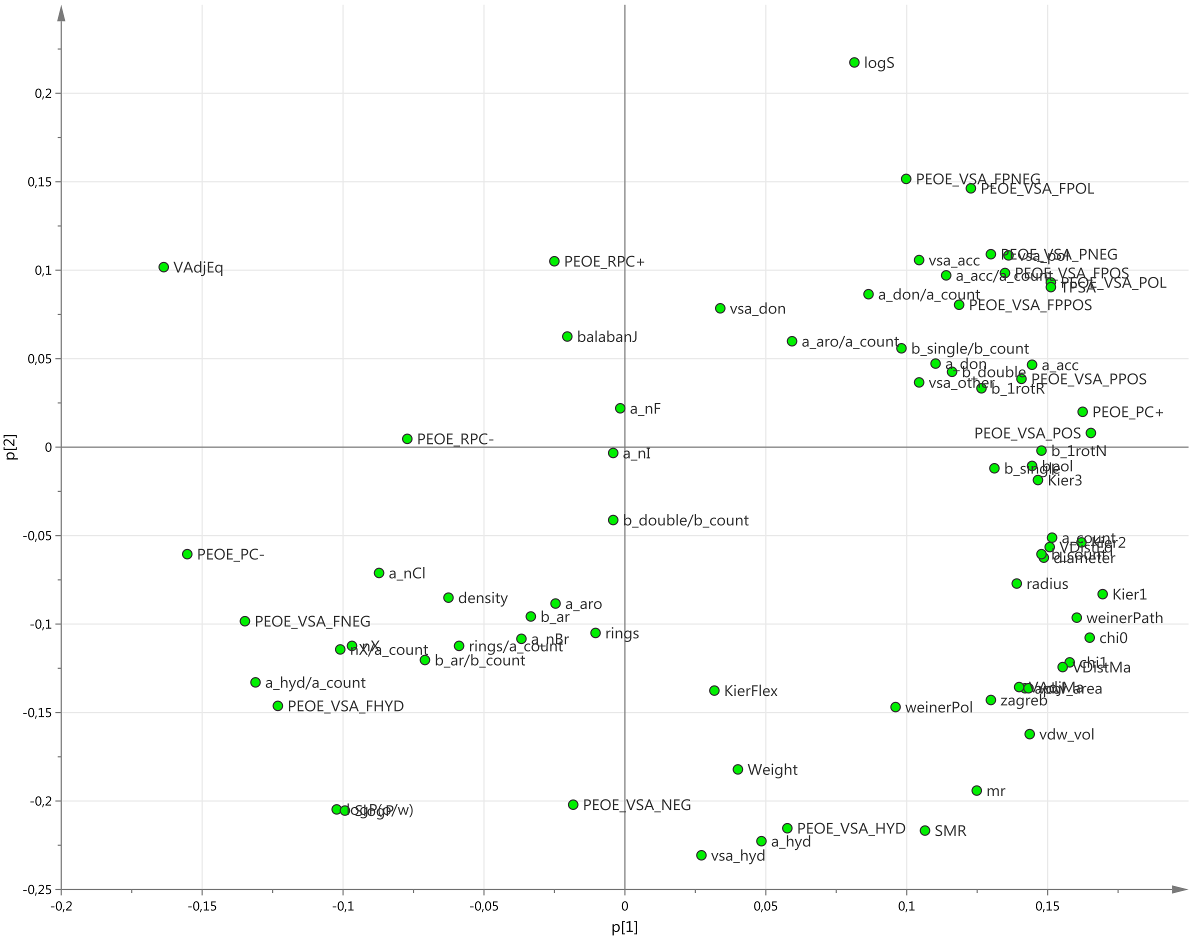

Figure S6b. The loading plot of the first and second (p_1 and p_2) principal components of the principal component analysis of the 429 industrial chemicals and the 214 AhR modulators (including the 66 AhR binders), explaining 38% and 18%, respectively, of the variation in the data.

1<br>18(18)<br>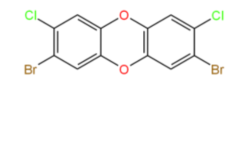	2<br>21(21)<br>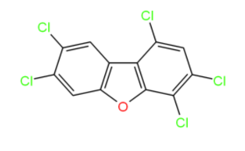	3<br>6(1)<br>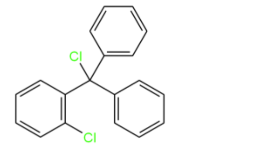	4<br>12(12)<br>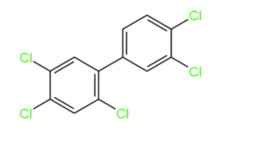	5<br>6(6)<br>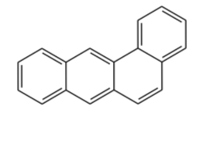
6<br>12(0)<br>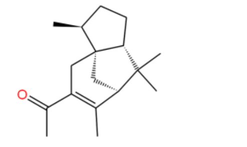	7<br>6(0)<br>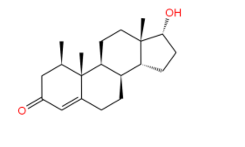	8<br>10(0)<br>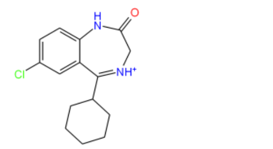	9<br>2(0)<br>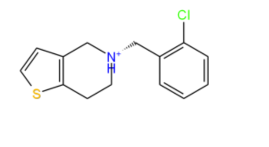	10<br>10(0)<br>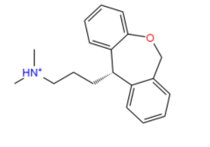
11<br>4(0)<br>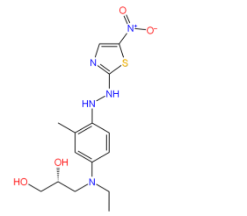	12<br>6(0)<br>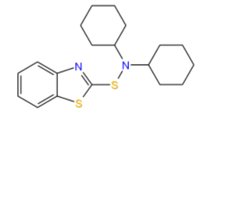	13<br>4(0)<br>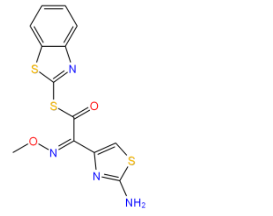	14<br>4(1)<br>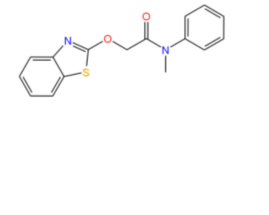	15<br>9(1)<br>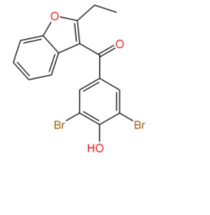
16<br>7(2)<br>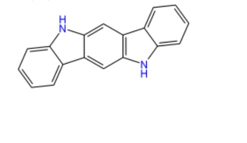	17<br>4(0)<br>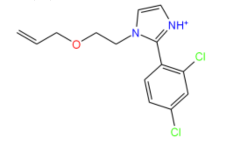	18<br>6(0)<br>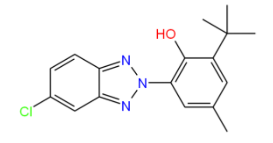	19<br>4(0)<br>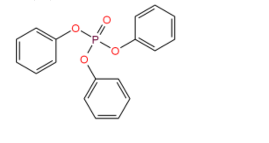	20<br>2(0)<br>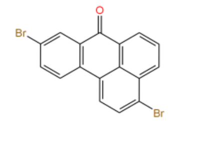
21<br>7(0)<br>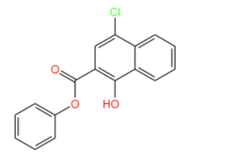	22<br>4(0)<br>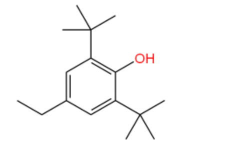	23<br>3(0)<br>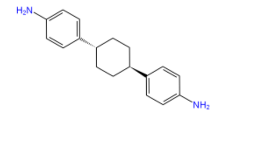	24<br>4(0)<br>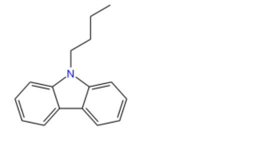	25<br>8(0)<br>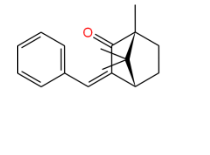
26<br>6(0)<br>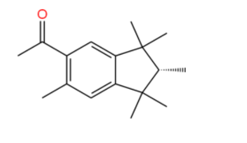	27<br>3(0)<br>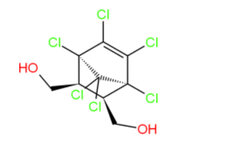	28<br>3(0)<br>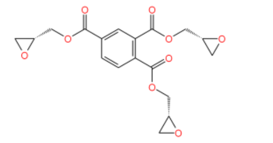	29<br>5(0)<br>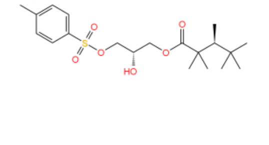	30<br>5(0)<br>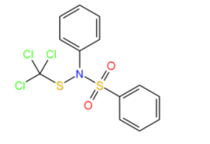
31<br>8(0)<br>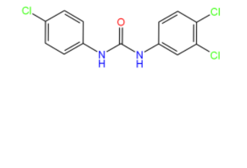	32<br>15(0)<br>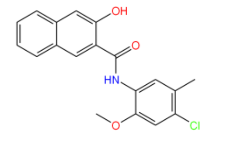	33<br>7(0)<br>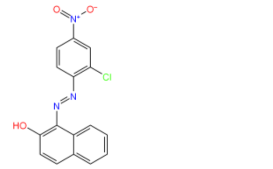	34<br>6(0)<br>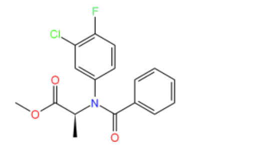	35<br>8(2)<br>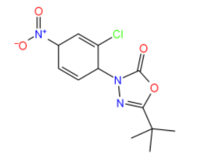

Figure S7. Clusters of compounds as a result of a hierarchical clustering where each frame represents a cluster, the cluster numbers are given in the upper left corner, the number of cluster members in the right corner with the number of known binders within brackets, and where the molecule was the centre of each cluster.

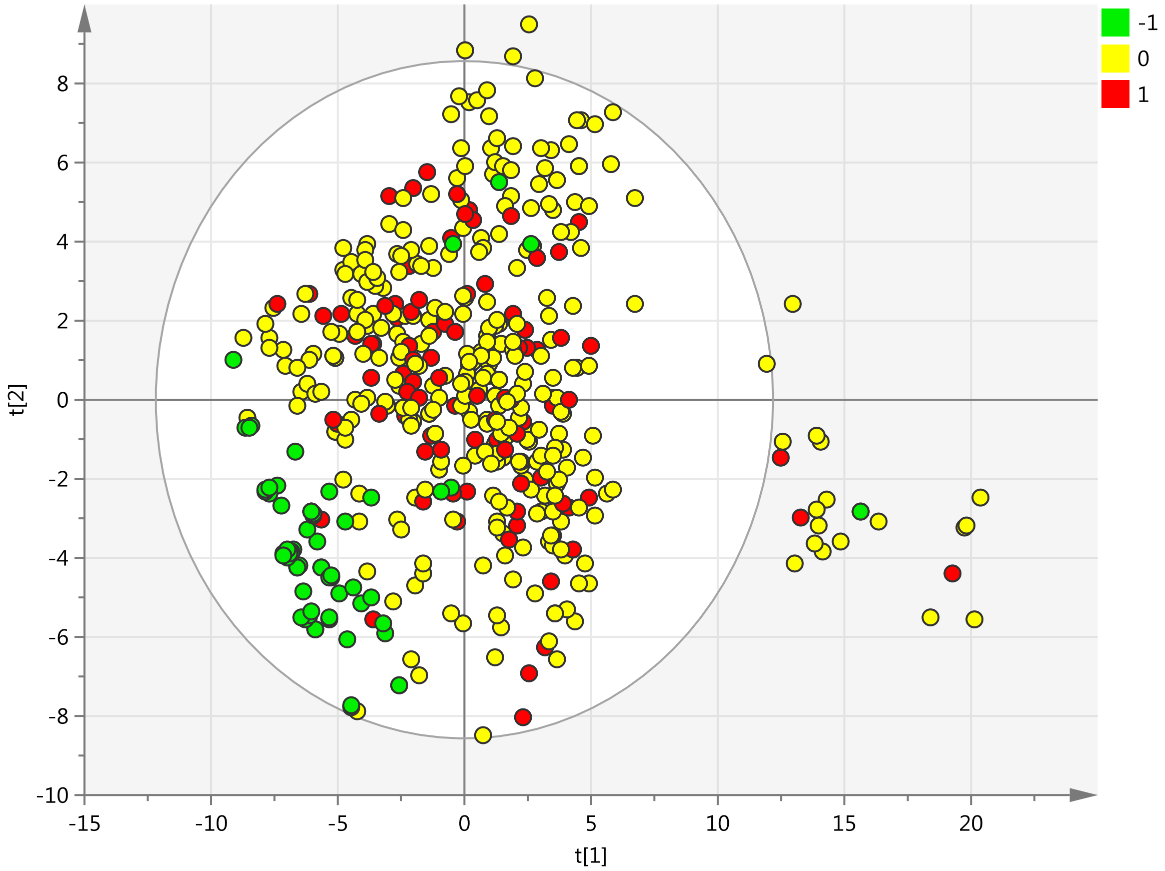

Figure S8a. The score plot of the principal component analysis of the 429 industrial chemicals screened in the parallel steps and the 66 AhR binders. The AhR binders are here depicted as green dots, the compounds tested within Tox21 are depicted as red dots, and the remaining chemicals as yellow dots. The variation in the PCs is 36 % and 18 % respectively.

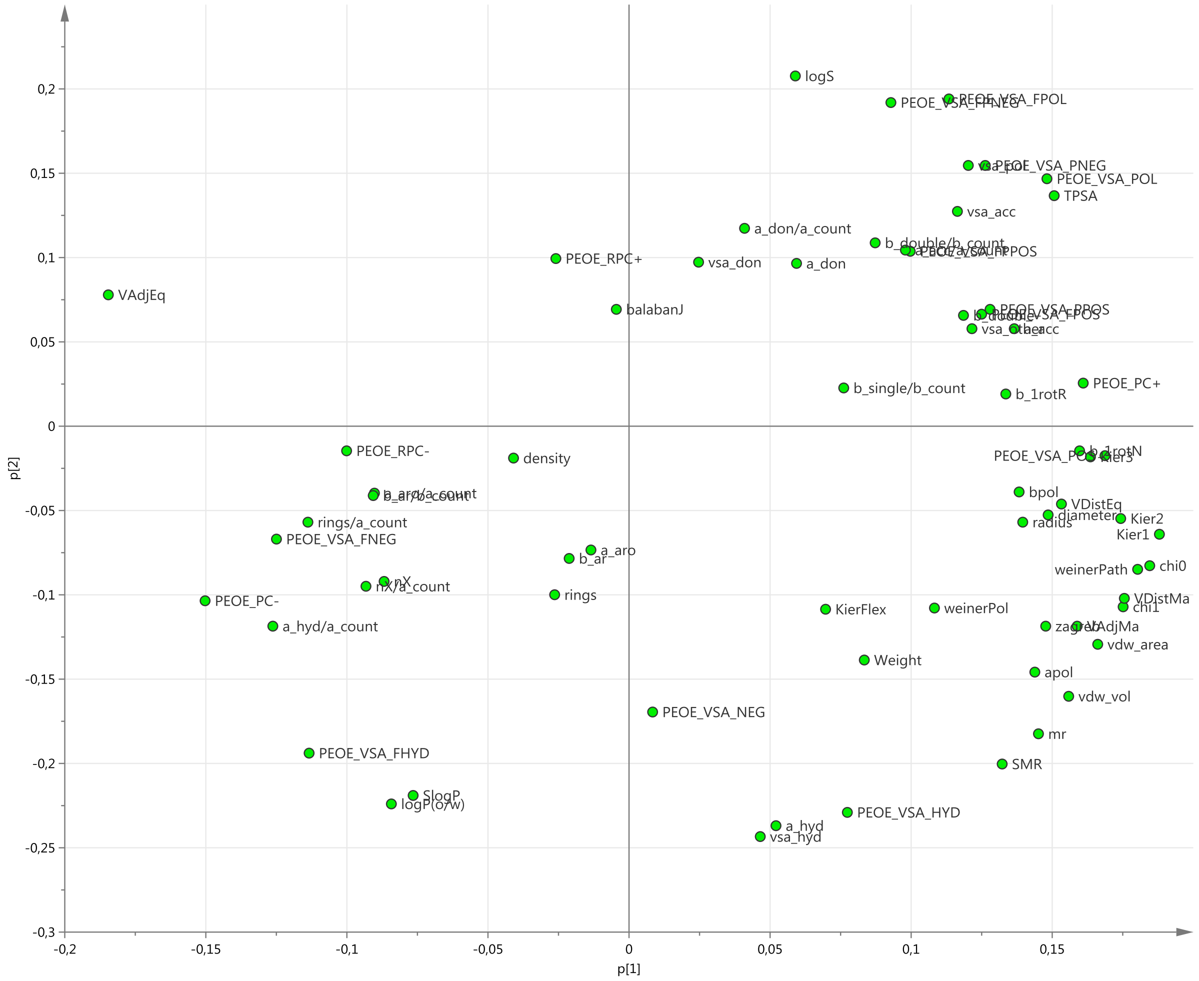

Figure S8b. The loading plot of the first and second (p_1 and p_2) principal components of the principal component analysis of the 429 industrial chemicals and the 66 AhR binders, explaining 36% and 18%, respectively, of the variation in the data.

Tables

Table S1. The 3D descriptors used in the principal component analysis of the 214 AhR modulators. The following descriptors depend on the structure connectivity and conformation (dimensions are measured in Ångstrom) (MOE 2012.10).

Descriptor	Description	Characterization
AM1_dipole	The dipole moment calculated using the AM1 Hamiltonian (Dewar et al. 1985).	polarizability
AM1_HOMO	The energy (eV) of the Highest Occupied Molecular Orbital (HOMO) calculated using the AM1 Hamiltonian (Dewar et al. 1985).	reactivity
AM1_LUMO	The energy (eV) of the Lowest Unoccupied Molecular Orbital (LUMO) calculated using the AM1 Hamiltonian (Dewar et al. 1985).	reactivity
AM1_GAP	Difference in LUMO and HOMO energy (LUMO-HOMO, eV) calculated using the AM1 Hamiltonian (Dewar et al. 1985).	reactivity
ASA	Water accessible surface area calculated using a radius of 1.4 Å for the water molecule. A polyhedral representation is used for each atom in calculating the surface area.	surface area characteristics
dens	Mass density: molecular weight divided by van der Waals volume as calculated in the vol descriptor.	shape
npr2	Normalized PMI ratio pmi2/pmi3 (Sauer et al 2003).	shape
pmi1	First diagonal element of diagonalized moment of inertia tensor (Sauer et al 2003).	size
pmi2	Second diagonal element of diagonalized moment of inertia tensor (Sauer et al 2003).	size
pmi3	Third diagonal element of diagonalized moment of inertia tensor (Sauer et al 2003).	size
std_dim1	Standard dimension 1: the square root of the largest eigenvalue of the covariance matrix of the atomic coordinates. A standard dimension is equivalent to the standard deviation along a principal component axis.	size
std_dim2	Standard dimension 2: the square root of the second largest eigenvalue of the covariance matrix of the atomic coordinates. A standard dimension is equivalent to the standard deviation along a principal component axis.	size
std_dim3	Standard dimension 3: the square root of the third largest eigenvalue of the covariance matrix of the atomic coordinates. A standard dimension is equivalent to the standard deviation along a principal component axis.	size
vol	van der Waals volume calculated using a grid approximation (spacing 0.75 Å).	size
VSA	van der Waals surface area. A polyhedral representation is used for each atom in calculating the surface area.	size
DistMax	DistMax calculates the longest distance (Ångstrom) between two atoms in the molecule from its 3D-coordinates (AM1-optimized) (Dewar et al. 1985).	size
pmi2/pmi1	Second diagonal element of diagonalized moment of inertia tensor divided by the first diagonal element of diagonalized moment of inertia tensor (Dewar et al. 1985).	shape
pmi3/pmi1	Third diagonal element of diagonalized moment of inertia tensor divided by the first diagonal element of diagonalized moment of inertia tensor (Dewar et al. 1985).	shape

Table S2. Characteristics of the clusters found in the principal component analysis (PCA) of the 214 AhR modulators.

**Cluster**[a]	**Characteristics**[b]
**Halogenated aromatics**<br>**Example:**<br>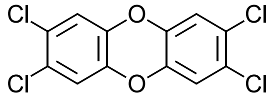<br>**2378-tetrachlorodibenzo-p-dioxin (2378-TCDD)**	Big hydrophobic surface area (PEOE_VSA_FHYD), high octanol-water partition coefficient (log P), big aromatic system compared to total number of atoms (a_aro/a_count).<br>Compared to PAHs: higher density (density), higher highest occupied molecular orbital (HOMO) energy (AM1_ HOMO), higher amount of branching (balabanJ).
**Polycyclic Aromatic Hydrocarbons**<br>**Example:**<br>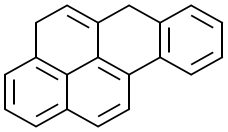<br>**benzo-a-pyrene (BaP)**<br>**Machala et al. 2001**	Big hydrophobic surface area (PEOE_VSA_FHYD), high octanol-water partition coefficient (log P), big aromatic system compared to total number of atoms (a_aro/a_count).<br>Compared to halogenated aromatics: lower reactivity as reflected by a larger difference between the LUMO and HOMO energies, i.e. large GAP (AM1_GAP), larger number of rings (rings).
**Endogenous compounds**<br>**Example:**<br>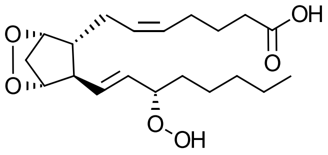<br>**prostaglandin G2**<br>**Seidel et al. 2001**	Big positive and polar surface area (PEOE_VSA_FPOS, PEOE_VSA_FPOL), many hydrogen acceptors and donors (a_don/a-count, a_acc/a_count), no aromatic system (a_aro/a_count), elongated structures (std_dim1, DistMax), long aliphatic chains/many rotatable bonds (b_1rotN, b_1rotR), high flexibility (KierFlex)<br>Compared to natural products: higher amount of branching (balabanJ) and higher flexibility (KierFlex), lack of aromatic system or rings (a_aro/a_count, rings).
**Natural products**<br>**Example:**<br>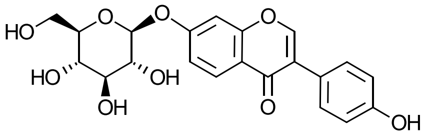<br>**daidzin**<br>**Amakura et al. 2011**	Big positive and polar surface area (PEOE_VSA_FPOS, PEOE_VSA_FPOL), many hydrogen acceptors and donors (a_don/a-count, a_acc/a_count), long aliphatic chains/many rotatable bonds, high amount of branching<br>Compared to endogenous compounds: aromatic compounds (a_aro), higher amount of branching (zagreb), and polarity calculated by topological index (weinerPol).

[a]Defined clusters by the PCA of the first, second and third principal component (Figure S3a).

[b]Interpretation of the PCA (Figure S3ab) using descriptors further explained in Section 2.5, Table S1 and by Stenberg et al.

Table S3. Structures of the industrial chemicals discussed and compared with data from the literature in Section 3.2.2 in the main text.

**Industrial chemical**	**AhR modulator from the literature**
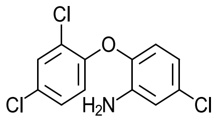 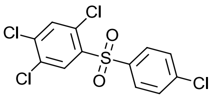 56966-52-0 116-29-0	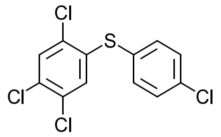 2,4,4',5-tetrachlorodiphenyl sulphide (Zhang et al. 2016)
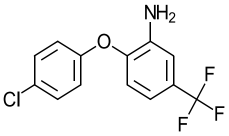 349-20-2	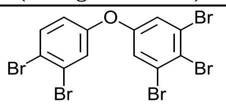 33'44'5-pentabromodiphenyl ether (Behnisch et al. 2003)
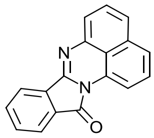 6925-69-5	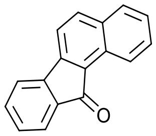 11H-benzo[a]fluorene-11-one (Bekki et al. 2009)
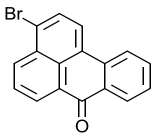 81-96-9	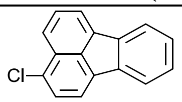 3-chlorofluoroanthene (Ohura et al. 2007) 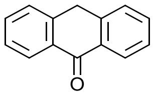 anthrone (Bekki et al. 2009)
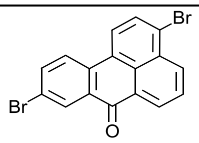 81-98-1	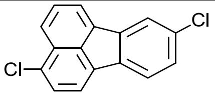 3,8-dichlorofluoroanthene (Ohura et al. 2007)
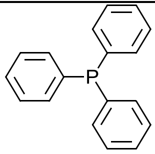 603-35-0	No similar compound found, but some resemblance with PAHs
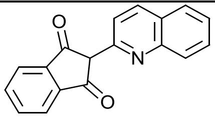 83-08-09	No similar compound found but some resemblance with 6-formylindolo[3,2-*b*]carbazole (FICZ) (Rannug et al. 1995)
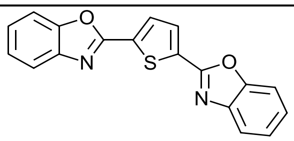 2866-43-5 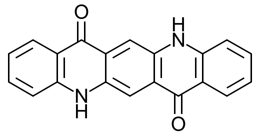 1047-16-1	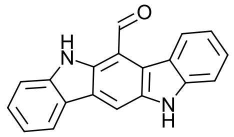 6-formylindolo[3,2-*b*]carbazole (FICZ) (Rannug et al. 1995)

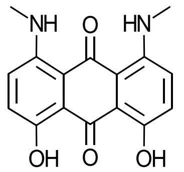<br>56524-76-6	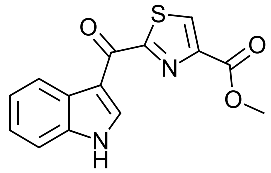<br>2-(1′*H*-indole-3′-carbonyl)-thiazole-4-carboxylic acid methyl ester (ITE) (Henry et al. 2006)
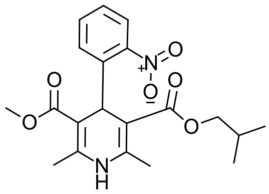<br>63675-72-9	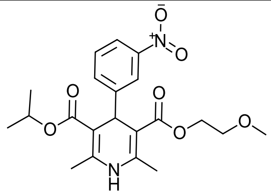<br>nimodipine (Hu et al. 2007)
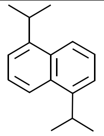<br>38640-62-9	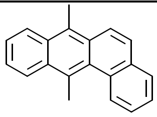<br>7,12-dimethyl-benzo-a-anthrazene (Machala et al. 2001)
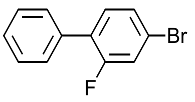<br>41604-19-7	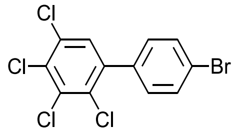<br>4’-bromo-2,3,4,5-tetrachloro-biphenyl (Bandiera et al. 1983)
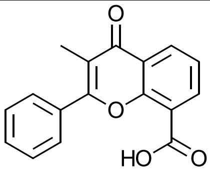<br>3468-017	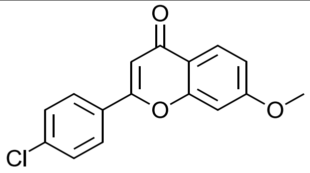<br>2-(4-chlorophenyl)-7-methoxy-4H-chromen-4-one (Chr-13) (Wall et al. 2012)
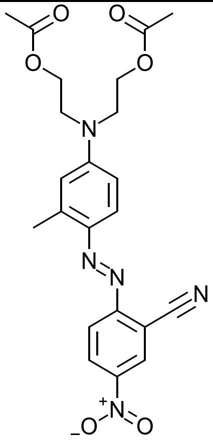<br>66882-16-4	No similar compound found but some resemblance with<br>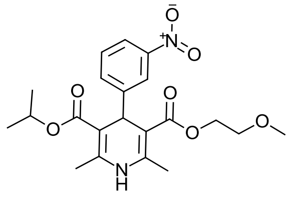<br>nimodipine (Hu et al. 2007)

Table S4. Structures of the industrial chemicals discussed and compared with data from the literature in Section 3.2.3 in the main text.

**Industrial chemical**	**AhR modulator from literature**
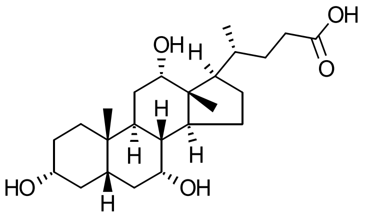 81-25-4	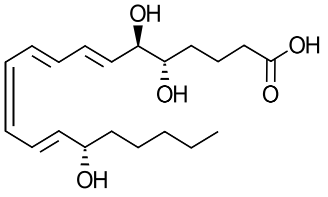 lipoxin 4A (Schaldach et al. 1999) 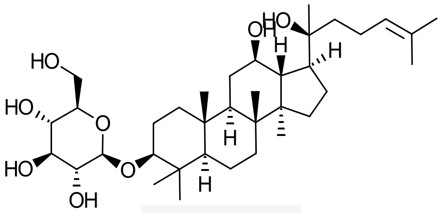 ginsenoside Rh2 (Hu et al. 2013)
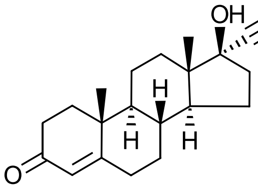 434-03-7	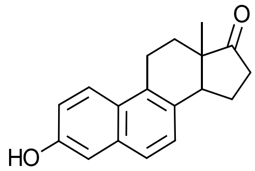 equilenin (Jinno et al. 2006)
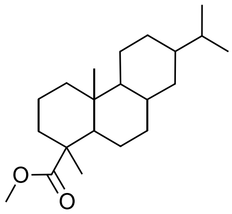 19941-28-7	No similar compound found, but some resemblance with equilenin (above)
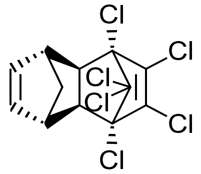 309-00-2 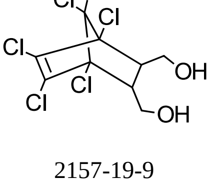 2157-19-9	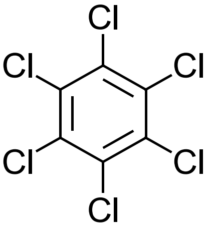 hexachlorobenzene (HCB) (de Tomaso Portaz et al. 2015)
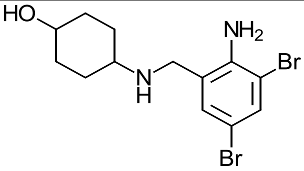	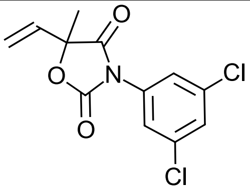

18683-91-5	vinclozolin (Oca et al. 2015)
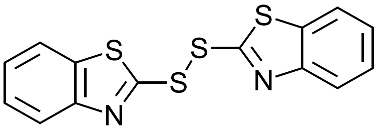<br>120-78-5	Already included in the study by He et al. 2011<br>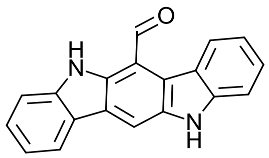<br>FICZ (Rannug et al. 1995)
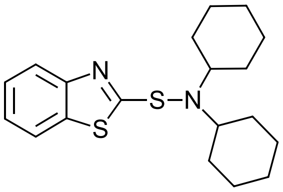<br>4979-32-2<br>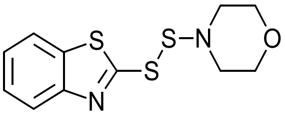<br>95-32-9<br>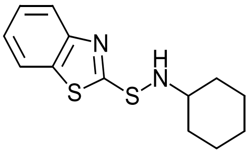<br>95-33-0<br>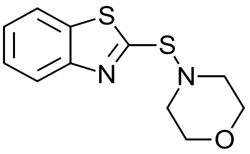<br>102-77-2	Already included in the study by He et al. 2011<br>Already included in the study by He et al. 2011<br>Already included in the study by He et al. 2011<br>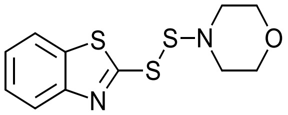<br>2-(morpholinodithio)benzothiazole (He et al. 2011)

Table S5. The structures of the 41 industrial chemicals that were identified as potential AhR ligands.

CAS number	Chemical name	Structure	Class[a]
31399-83-4	3-(2-chloro-4-nitrophenyl)-5-(1,1-dimethylethyl)-1,3,4-oxadiazol-2(3H)-one	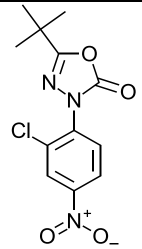	1
32534-81-9	2,2',4,4',5-pentabromodiphenylether (BDE99)	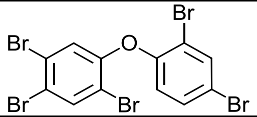	1
3380-34-5	triclosan	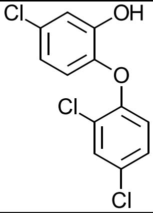	1
42074-68-0	2-chlorotrityl chloride	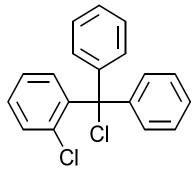	1
86-72-6	3-p-hydroxyaniline carbazole	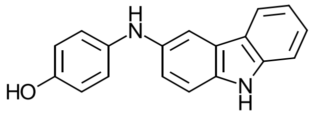	1
50-29-3	p,p'-dichlorodiphenyltrichloroethane (pp'-DDT)	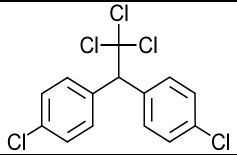	1
63675-72-9	nisoldipine	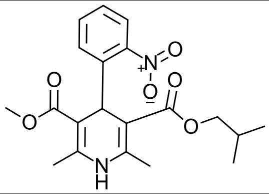	1
3896-11-05	bumetrizole	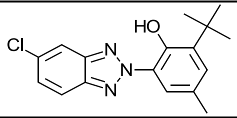	2SL
7643-08-05	2-methylthiophenothiazine	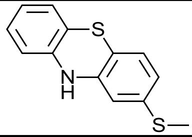	2SL
1041-00-5	2,2'-vinylene bis[5-methylbenzoxazole]	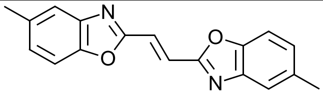	2SL
1047-16-1	5,12-dihydroquino[2,3-b]acridine-7,14-dione	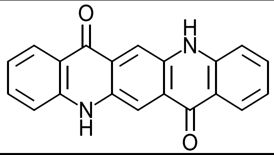	2SL

115-32-2	dicofol	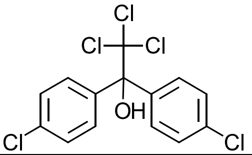	2SL
120-78-5	di(benzothiazol-2-yl) disulphide	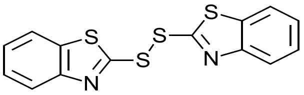	2SL
132-68-3	3-hydroxy-N-1-naphthyl-2-naphthamide	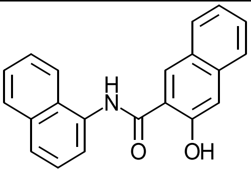	2SL
2392-48-5	4-chloro-1-(2,4-dichlorophenoxy)-2-nitrobenzene	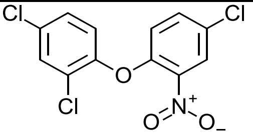	2SL
2475-46-9	1-methylamino-4-ethanolaminoanthraquinone (C.I. Disperse Blue 3)	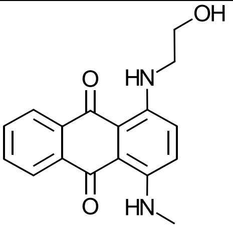	2SL
2832-40-8	N-[4-[(2-hydroxy-5-methylphenyl)azo]phenyl]acetamide	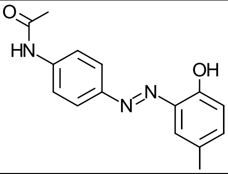	2SL
2866-43-5	2,2'-thiophene-2,5-diylbis(benzoxazole)	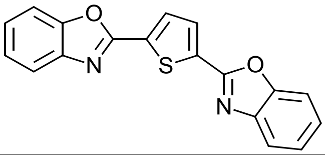	2SL
2879-15-4	8-benzyl-3,7-dihydro-1,3-dimethyl-1H-purine-2,6-dione	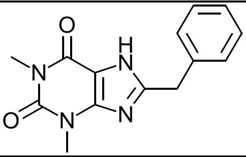	2SL
56966-52-0	5-chloro-2-(2,4-dichlorophenoxy) aniline	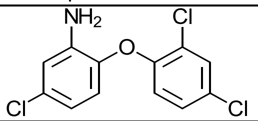	2SL
58979-46-7	N-[5-(diethylamino)-2-[(3,5-dinitro-2-thienyl)azo]phenyl]acetamide	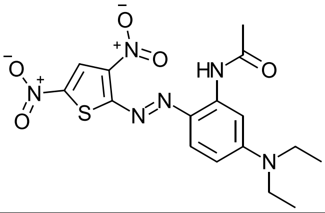	2SL
76-83-5	chlorotriphenylmethane	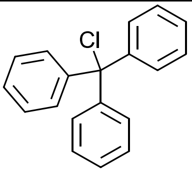	2SL

81-98-1	3,9-dibromo-7H-benz[de]anthracen-7-one	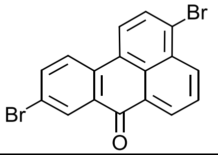	2SL
94279-09-1	2-hydroxy-3-[[(4-methylphenyl) sulphonyl]oxy]propyl tert-decanoate	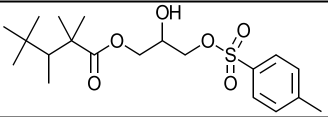	2SL
94279-10-4	3-hydroxy-2-[[(4-methylphenyl) sulphonyl]oxy]propyl tert-decanoate	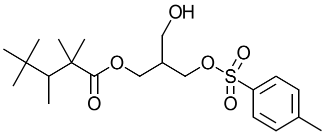	2SL
116-29-0	tetradifon	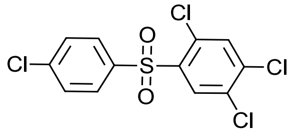	2SL
25059-80-7	ethyl 4-chloro-2-oxo-2H-benzo thiazole-3-acetate	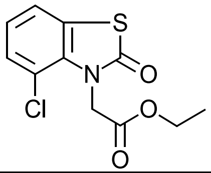	2SL
50471-44-8	N-3,5-dichlorophenyl-5-methyl-5-vinyl-1,3-oxazolidine-2,4-dione	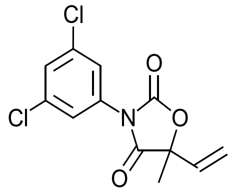	2SL
5495-84-1	2-isopropyl-9H-thioxanthen-9-one	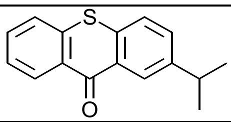	2SL
15307-93-4	2,6-dichloro-N-phenylaniline	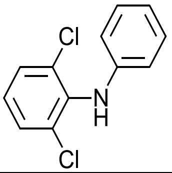	2L
1775-95-7	2-amino-5-nitrobenzophenone	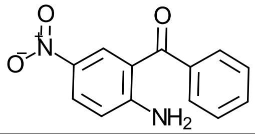	2L
24112-48-9	N-[5-(diethylamino)-2-[(2,4-dinitrophenyl)azo]phenyl]acetamide	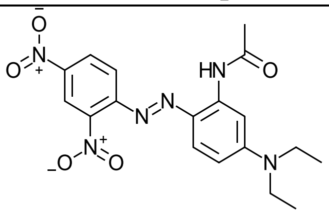	2L
26140-60-3	terphenyl	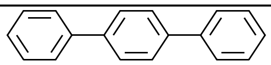	2L
38982-12-6	3-(9-anthryl)acrylaldehyde	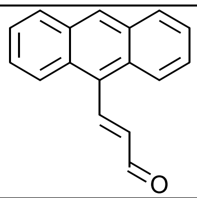	2L

41604-19-7	4-bromo-2-fluoro-1,1'-biphenyl	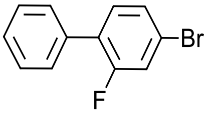	2L
41642-51-7	N-[2-[(2,6-dicyano-4-nitrophenyl) azo]-5-(diethylamino) phenyl] acetamide	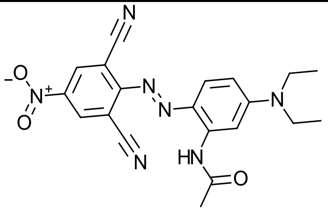	2L
59383-11-8	2-chloroethyl 3-nitro-p-toluate	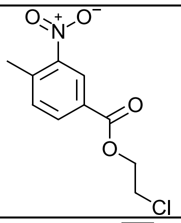	2L
603-35-0	triphenylphosphine	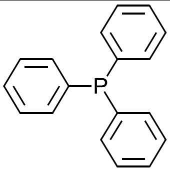	2L
642-31-9	anthracene-9-carbaldehyde	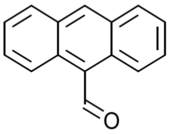	2L
81-37-8	2,8-dimethylnaphtho[3,2,1-kl]xanthene	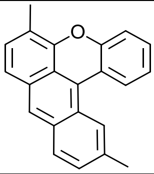	2L
97-32-5	4-methoxy-3-nitro-N-phenylbenzamide	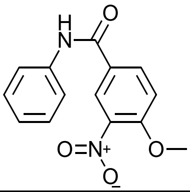	2L

[a] Chemicals that were identified by all three enrichment methods are marked with with “1” and the chemicals that were identified by two but not three of the enrichments methods are marked with “2”. SL stands for when a ligand-based method and a structure-based method identified the same chemical, and L stands for when the two ligand-based methods identified the same chemical.

Table S6. Registration information of the compounds (among the 41 jointly identified potential AhR ligands) that are registered in REACH [2015-05-28].

CAS number	Substance name	Production volume / registration information
42074-68-0	1-chloro-2-(chlorodiphenylmethyl)benzene / 2-chlorotritylchloride	Intermediate[a]
3380-34-5	triclosan	1000 – 10000 tonnes / year
15307-93-4	2,6-dichloro-N-phenylaniline	Intermediate[a]
38982-12-6	3-(9-anthryl)acrylaldehyde	Intermediate[a]
642-31-9	anthracene-9-carbaldehyde	Intermediate[a]
26140-60-3	terphenyl	Intermediate[a]
603-35-0	triphenylphosphine	100+ tonnes /year
1047-16-1	5,12-dihydroquino[2,3-b]acridine-7,14-dione	1000 – 10 000 tonnes / year
56966-52-0	5-chloro-2-(2,4-dichlorophenoxy)aniline	Intermediate[a]
3896-11-5	bumetrizole	100 – 1000 tonnes / year
120-78-5	di(benzothiazol-2-yl) disulphide	10 000 – 100 000 tonnes / year

[a]Compounds registered for intermediate use are to be isolated in the chemical processing and transformed into another substance, either on the same site as the final product or transported between or supplied to other sites (ECHA).

Table S7. AhR-related response data for compounds among the 41 jointly identified potential AhR ligands found by search in SciFinder [2016-03-16].

CAS number	Substance name	AhR-mediated activity	Reference
115-32-2	dicofol	Yes	Chan et al. 2009
120-78-5	di(benzothiazol-2-yl) disulphide	Yes	He et al. 2011
32534-81-9	2,2',4,4',5-pentabromodiphenylether (BDE99)	Yes	Hamers et al. 2006
3380-34-5	triclosan	Yes	Ahn et al. 2008
			Fent et al. 2014,
3896-11-5	bumetrizole	Yes	Nagayoshi et al. 2015
50-29-3	clofenotane/ p,p'-dichlorodiphenyl-trichloroethane (pp'-DDT)	Yes	Wojtowicz et al. 2011
50471-44-8	vinclozolin	Yes	Oca et al. 2015
5495-84-1	2-isopropylthioxaanthone	Yes	Peijnenburg et al. 2010
642-31-9	anthracene-9-carbaldehyde	No	Takeushi et al. 2008

Table S8. Overview of tested AhR activity based on ToxCast and PubChem Bioassay databases within Tox21 for the 41 jointly identified potential AhR ligands.

CAS number	Substance name	PubChem Bioassay[a]	Prioritized chemicals[d]
1041-00-5	2,2'-vinylenebis[5-methylbenzoxazole]	--[b]	X
1047-16-1	5,12-dihydroquino[2,3-b]acridine-7,14-dione	--[b]	X
115-32-2	dicofol	--[c]	Chan et al.
116-29-0	tetradifon	**Inactive**<br>**Inconclusive antagonist**	
120-78-5	di(benzothiazol-2-yl) disulphide	--[c]	He et al.
132-68-3	3-hydroxy-N-1-naphthyl-2-naphthamide	--[b]	X
15307-93-4	2,6-dichloro-N-phenylaniline	--[b]	X
1775-95-7	2-amino-5-nitrobenzophenone	--[b]	X
2392-48-5	4-chloro-1-(2,4-dichlorophenoxy)-2-nitrobenzene	--[b]	X
24112-48-9	N-[5-(diethylamino)-2-[(2,4-dinitrophenyl)azo]phenyl]acetamide	--[b]	X
2475-46-9	C.I. Disperse Blue 3/ 1-methylamino-4-ethanolaminoanthraquinone	--[b]	X
25059-80-7	ethyl 4-chloro-2-oxo-2H-benzothiazole-3-acetate	**Active agonist**	
26140-60-3	terphenyl	--[b]	X
2832-40-8	N-[4-[(2-hydroxy-5-methylphenyl)azo]phenyl]acetamide	--[b]	X
2866-43-5	2,2'-thiophene-2,5-diylbis(benzoxazole)	--[b]	X
2879-15-4	8-benzyl-3,7-dihydro-1,3-dimethyl-1H-purine-2,6-dione	--[b]	X
31399-83-4	3-(2-chloro-4-nitrophenyl)-5-(1,1-dimethylethyl)-1,3,4-oxadiazol-2(3H)-one	--[b]	X
32534-81-9	2,2’,4,4’,5-pentabromodiphenyl ether (BDE99)	--[c]	Hamers et al.
3380-34-5	triclosan	**Inconclusive agonist**	
3896-11-05	bumetrizole	--[b]	
38982-12-6	3-(9-anthryl)acrylaldehyde	--[b]	X
41604-19-7	4-bromo-2-fluoro-1,1'-biphenyl	--[b]	X
41642-51-7	N-[2-[(2,6-dicyano-4-nitrophenyl)azo]-5-(diethylamino)phenyl]acetamide	--[b]	X
42074-68-0	1-chloro-2-(chlorodiphenylmethyl)benzene	--[b]	X
50-29-3	clofenotane / p,p'-dichlorodiphenyltrichloroethane (pp’-DDT)	Inactive	
50471-44-8	N-3,5-dichlorophenyl-5-methyl-5-vinyl-1,3-oxazolidine-2,4-dione	Inactive	
5495-84-1	2-isopropyl-9H-thioxanthen-9-one	**Active agonist**	
56966-52-0	5-chloro-2-(2,4-dichlorophenoxy)aniline	--[b]	X
58979-46-7	N-[5-(diethylamino)-2-[(3,5-dinitro-2-thienyl)azo]phenyl]acetamide	--[b]	X
59383-11-8	2-chloroethyl 3-nitro-p-toluate	--[b]	X
603-35-0	triphenylphosphine	--[c]	X
63675-72-9	nisoldipine	**Active agonist**	
642-31-9	anthracene-9-carbaldehyde	--[b]	Takeushi et al.
7643-08-05	2-methylthiophenothiazine	--[b]	X
76-83-5	chlorotriphenylmethane	--[b]	X

81-37-8	2,8-dimethylnaphtho[3,2,1-kl]xanthene	--[b]	X
81-98-1	3,9-dibromo-7H-benz[de]anthracen-7-one	--[b]	X
86-72-6	4-(9H-carbazol-3-ylamino)phenol	--[b]	X
94279-09-1	2-hydroxy-3-[[(4-methylphenyl)sulphonyl]oxy]propyl tert-decanoate	--[b]	X
94279-10-4	3-hydroxy-2-[[(4-methylphenyl)sulphonyl]oxy]propyl tert-decanoate	--[b]	X
97-32-5	4-methoxy-3-nitro-N-phenylbenzamide	**Active agonist**	

[a] https://ntp.niehs.nih.gov/sandbox/tox21-curve-visualization/ (accessed Jan 26, 2017). The studies used 14 concentrations in the range 0.01-10uM.

[b] Not tested, the compound was not included in the studied database.

[c] Tested in Tox21 but with a purity less than 90%

[d] Compounds not tested for AhR activity according to our scientific literature search or tested in Tox21 at a purity less than 90% or with lacking purity information.

Table S9. Comparisons of 94 compounds tested in the PubChem Bioassay and the enrichments from the parallel steps.

**Number of compounds**	**Structural fingerprints**	**Nearest Neighbor analysis**	**Molecular docking**
True Positives (TP)	2	9	20
True Negatives (TN)	55	50	30
False Positives (FP)	1	6	26
False Negatives (FN)	36	29	20
Accuracy =(TP+TN)/(TP+FP+FN+TN)	0.61	0.63	0.53
Sensitivity = TP/(TP+FN)	0.05	0.24	0.53
Specificity = TN/(TN+FP)	0.98	0.89	0.54

References

Ahn KC et al. (2008) In vitro biologic activities of the antimicrobials triclocarban, its analogs, and triclosan in bioassay screens: Receptor-based bioassay screens Environ Health Perspect 116:1203-1210 doi:10.1289/ehp.11200

Bandiera S, Sawyer TW, Campbell MA, Fujita T, Safe S (1983) Competitive-binding to the cytosolic 2,3,7,8-tetrachlorodibenzo-para-dioxin receptor - effects of structure on the affinities of substituted halogenated biphenyls - a QSAR analysis Biochem Pharmacol 32:3803-3813 doi:10.1016/0006-2952(83)90153-3

Behnisch PA, Hosoe K, Sakai S-i (2003) Brominated dioxin-like compounds: in vitro assessment in comparison to classical dioxin-like compounds and other polyaromatic compounds Environ Int 29:861-877 doi:10.1016/s0160-4120(03)00105-3

Bekki K, Takigami H, Suzuki G, Tang N, Hayakawa K (2009) Evaluation of toxic activities of polycyclic aromatic hydrocarbon derivatives using in vitro bioassays J Health Sci 55:601-610 doi:10.1248/jhs.55.601

Casado S, Alonso M, Herradon B, Tarazona JV, Navas J (2006) Activation of the aryl hydrocarbon receptor by carbaryl: Computational evidence of the ability of carbaryl to assume a planar conformation Environ Toxicol Chem 25:3141-3147 doi: 10.1897/06-131R.1
de Oca FGG-M, de Lourdes López-González M, Escobar-Wilches DC, Chavira-Ramírez R, Sierra-Santoyo A (2015) Vinclozolin modulates hepatic cytochrome P450 isoforms during pregnancy Reprod Toxicol 53:119-126 doi:10.1016/j.reprotox.2015.04.010

de Tomaso Portaz AC, Caimi GR, Sánchez M, Chiappini F, Randi AS, de Pisarev DLK, Alvarez L (2015) Hexachlorobenzene induces cell proliferation, and aryl hydrocarbon receptor expression (AhR) in rat liver preneoplastic foci, and in the human hepatoma cell line HepG2. AhR is a mediator of ERK1/2 signaling, and cell cycle regulation in HCB-treated HepG2 cells Toxicol 336:36-47 doi:10.1016/j.tox.2015.07.013

Denison MS, Nagy SR (2003) Activation of the aryl hydrocarbon receptor by structurally diverse exogenous and endogenous chemicals Annu Rev Pharmacolog 43:309-334 doi:10.1146/annurev.pharmtox.43.100901.135828

Dewar MJS, Zoebisch EG, Healy EF, Stewart JJP (1985) The Development and Use of Quantum-Mechanical Molecular-Models .76. Am1 - a New General-Purpose Quantum-Mechanical Molecular-Model J Am Chem Soc 107 3902-3909 doi:10.1021/ja00299a024

ECHA Guidance on intermediates Version 2, December 2010. Available at http://echa.europa.eu/documents/10162/13632/intermediates_en.pdf [2015-04-23].

Fent K, Chew G, Li J, Gomez E (2014) Benzotriazole UV-stabilizers and benzotriazole: Antiandrogenic activity in vitro and activation of aryl hydrocarbon receptor pathway in zebrafish eleuthero-embryos Sci Total Environ 482-483:125-136 doi:10.1016/j.scitotenv.2014.02.109

Garrison PM, Tullis K, Aarts J, Brouwer A, Giesy JP, Denison MS (1996) Species-specific recombinant cell lines as bioassay systems for the detection of 2,3,7,8-tetrachlorodibenzo-p-dioxin-like chemicals Fundamental and Applied Toxicology 30:194-203 doi:10.1006/faat.1996.0056

Hamers T et al. (2006) In vitro profiling of the endocrine-disrupting potency of brominated flame retardants Toxicol Sci 92:157-173 doi:10.1093/toxsci/kfj187

He G, Zhao B, Denison MS (2011) Identification of benzothiazole derivatives and polycyclic aromatic hydrocarbons as aryl hydrocarbon receptor agonists present in tire extracts Environ Toxicol Chem 30:1915-1925 doi:10.1002/etc.581

Henry EC, Bemis JC, Henry O, Kende AS, Gasiewicz TA (2006) A potential endogenous ligand for the aryl hydrocarbon receptor has potent agonist activity in vitro and in vivo Arch Biochem Biophys 450:67-77 doi:10.1016/j.abb.2006.02.008

Henry EC et al. (1999) Flavone antagonists bind competitively with 2,3,7,8-tetrachlorodibenzo-p-dioxin (TCDD) to the aryl hydrocarbon receptor but inhibit nuclear uptake and transformation Mol Pharmacol 55:716-725

Hu Q et al. (2013) Ginsenosides are novel naturally-occurring aryl hydrocarbon receptor ligands PLoS One 8:e66258 doi:10.1371/journal.pone.0066258

Hu W, Sorrentino C, Denison MS, Kolaja K, Fielden MR (2007) Induction of Cyp1a1 is a nonspecific biomarker of aryl hydrocarbon receptor activation: Results of large scale screening of pharmaceuticals and toxicants in vivo and in vitro Mol Pharm 71:1475-1486 doi:10.1124/mol.106.032748

Jinno A, Maruyama Y, Ishizuka M, Kazusaka A, Nakamura A, Fujita S (2006) Induction of cytochrome P450-1A by the equine estrogen equilenin, a new endogenous aryl hydrocarbon receptor ligand J Steroid Biochem Mol Biol 98:48-55 doi:10.1016/j.jsbmb.2005.07.003

Larsson M, Kumar Mishra B, Tysklind M, Linusson A, Andersson PL (2013) On the use of electronic descriptors for QSAR modelling of PCDDs, PCDFs and dioxin-like PCBs SAR QSAR Environ Res 24:461-479 doi:10.1080/1062936x.2013.791719

Machala M, Vondracek J, Blaha L, Ciganek M, Neca J (2001) Aryl hydrocarbon receptor-mediated activity of mutagenic polycyclic aromatic hydrocarbons determined using in vitro reporter gene assay Mutat Res,Gen Tox En 497:49-62 doi:10.1016/s1383-5718(01)00240-6

McKinney JD, Pedersen LG (1986) Biological-activity of polychlorinated biphenyls related to conformational structure Biochem J 240:621-622

Molecular operating environment (MOE) 2012.10. Chemical Computing Group, Quebec, Canada.

Nagayoshi H, Kakimoto K, Takagi S, Konishi Y, Kajimura K, Matsuda T (2015) Benzotriazole Ultraviolet Stabilizers Show Potent Activities as Human Aryl Hydrocarbon Receptor Ligands Environ Sci Technol 49:578-587 doi:10.1021/es503926w

Ohura T, Morita M, Makino M, Amagai T, Shimoi K (2007) Aryl hydrocarbon receptor-mediated effects of chlorinated polycyclic aromatic hydrocarbons Chem Res Toxicol 20:1237-1241 doi:10.1021/tx700148b

Peijnenburg A, Riethof-Poortman J, Baykus H, Portier L, Bovee T, Hoogenboom R (2010) AhR-agonistic, anti-androgenic, and anti-estrogenic potencies of 2-isopropylthioxanthone (ITX) as determined by in vitro bioassays and gene expression profiling Toxicol in Vitro 24:1619-1628 doi:10.1016/j.tiv.2010.06.004

Rannar S, Andersson PL (2010) A Novel Approach Using Hierarchical Clustering To Select Industrial Chemicals for Environmental Impact Assessment J Chem Inf Model 50:30-36 doi:10.1021/ci9003255

Rannug U, Rannug A, Sjoberg U, Li H, Westerholm R, Bergman J (1995) Structure elucidation of two tryptophan-derived, high affinity Ah receptor ligands Chem Biol 2:841-845 doi:10.1016/1074-5521(95)90090-x

Sauer WHB, Schwarz MK (2003) Molecular shape diversity of combinatorial libraries: A prerequisite for broad bioactivity J Chem Inf Comp Sci 43:987-1003 doi:10.1021/ci025599w

Schaldach CM, Riby J, Bjeldanes LF (1999) Lipoxin A(4): A new class of ligand for the Ah receptor Biochem 38:7594-7600 doi:10.1021/bi982861e

Seidel SD, Winters GM, Rogers WJ, Ziccardi MH, Li V, Keser B, Denison MS (2001) Activation of the Ah receptor signaling pathway by prostaglandins Journal of Biochemical and Molecular Toxicology 15:187-196 doi: 10.1002/jbt.16

Stenberg M, Linusson A, Tysklind M, Andersson PL (2009) A multivariate chemical map of industrial chemicals - Assessment of various protocols for identification of chemicals of potential concern Chemosphere 76:878-884 doi:10.1016/j.chemosphere.2009.05.011

Takeuchi S, Iida M, Yabushita H, Matsuda T, Kojima H (2008) In vitro screening for aryl hydrocarbon receptor agonistic activity in 200 pesticides using a highly sensitive reporter cell line, DR-EcoScreen cells, and in vivo mouse liver cytochrome P450-1A induction by propanil, diuron and linuron Chemosphere 74:155-165 doi:10.1016/j.chemosphere.2008.08.015

Wall RJ et al. (2012) Novel 2-amino-isoflavones exhibit aryl hydrocarbon receptor agonist or antagonist activity in a species/cell-specific context Toxicol 297:26-33 doi:10.1016/j.tox.2012.03.011

Van den Berg M et al. (2006) The 2005 World Health Organization Reevaluation of Human and Mammalian Toxic Equivalency Factors for Dioxins and Dioxin-Like Compounds Toxicol Sci 93:223-241 doi:10.1093/toxsci/kfl055

Wojtowicz AK, Honkisz E, Zieba-Przybylska D, Milewicz T, Kajta M (2011) Effects of two isomers of DDT and their metabolite DDE on CYP1A1 and AhR function in human placental cells Pharmacol Res 63:1460-1468 doi:10.1016/S1734-1140(11)70710-1

Zhang J et al. (2016) Activation of AhR-mediated toxicity pathway by emerging pollutants polychlorinated diphenyl sulfides Chemosphere 144:1754-1762 doi:10.1016/j.chemosphere.2015.09.107
